# Signed log-likelihood ratio test for the scale parameter of Poisson Inverse Weibull distribution with the development of PIW4LIFETIME web application

**DOI:** 10.1371/journal.pone.0329293

**Published:** 2025-08-01

**Authors:** Sukanya Yodnual, Jularat Chumnaul

**Affiliations:** Division of Computational Science, Faculty of Science, Prince of Songkla University, Hat Yai, Songkhla, Thailand; Cairo University, EGYPT

## Abstract

The three-parameter Poisson Inverse Weibull (PIW) distribution offers enhanced flexibility for modeling system failure times. This study introduces the signed log-likelihood ratio test (SLRT) for hypothesis testing of the scale parameter (ω) in the PIW distribution and compares its performance with the test based on the asymptotic normality of maximum likelihood estimators (ANMLE). Simulation studies show that the SLRT consistently maintains type I error rates within the acceptable range of 0.04 to 0.06 at a significance level of 0.05, satisfying Cochran’s criterion across various sample sizes and parameter configurations. In contrast, the ANMLE method tends to be conservative, often underestimating the nominal significance level. In terms of empirical power, the SLRT outperforms the ANMLE, particularly in small-sample scenarios (*n* = 10, 15), and maintains superior power across all tested configurations. For example, when testing $H_{0}:\omega =0.25$ against $H_{1}:\omega =0.5$ with β=0.5,λ=1, and *n* = 10, the SLRT achieves a power of 0.6621, compared to 0.4181 for the ANMLE, demonstrating the SLRT’s robustness and reliability in limited-data. Moreover, the ANMLE generally exhibits low power in most cases, indicating reduced sensitivity to detecting true effects in small samples. However, with medium and large sample sizes (*n* = 30, 50, 80 and 100), the power of the ANMLE begins to approach that of the SLRT. Despite this, the ANMLE never outperforms the SLRT, highlighting a fundamental limitation of this method. Additionally, varying the shape parameter β while fixing λ=1 showed a negligible impact on power, further confirming the robustness of the SLRT. Sensitivity analyses also validate the reliability of the SLRT under extreme values of ω and across different sample sizes. To support practical application, the PIW4LIFETIME web application (accessible at https://jularatchumnaul.shinyapps.io/PIW4LIFETIME/) was developed to enable users to assess whether data fit the PIW distribution, estimate model parameters using maximum likelihood, and perform two-sided test for the scale parameter using SLRT. The performance of the proposed method and the PIW4LIFETIME web application was demonstrated through a real-world example.

## Introduction

Statistical lifetime distributions are widely used in various research fields. For example, in actuarial sciences and insurance, researchers may study the duration of insurance policies to assess risk and analyze the period without claims on customer policies. In engineering, analyzing the usage duration of machinery and electrical equipment is crucial for predicting downtime or system failures. In medical sciences, researchers may focus on the survival times of patients after surgery or the duration until the recurrence of cancer post-surgery. In social sciences, researchers may study how long marriages last until divorce or the duration a graduate remains unemployed. In computer sciences, researchers might analyze the failure rates of software systems, whereas in marketing, they may assess customer lifetime values. For further details about the above lifetime applications, please refer to Lai and Xie (2006), Pham (2006), Pham (2007), Ohishi *et al*. (2009), Lai *et al*. (2011), Bemmaor and Glady (2012), Lai (2013), and Almalki (2014) [1–8].

When dealing with lifetime data, several common distributions are often used, including the exponential, gamma, lognormal, and Weibull distributions [9]. The Inverse Weibull distribution is also frequently applied in practical situations, such as analyzing the degradation of mechanical components (for example, pistons and crankshafts in diesel engines) and the breakdown of insulating fluids [10, 11]. It is also used in wind speed analysis [12] and the context of intuitive fuzzy life data [13]. Additionally, various extensions and modified versions of the Weibull distribution have been developed to enhance its ability to model and fit different sets of lifetime data [14].

Over the past ten years, many researchers have developed new statistical distributions derived from the Weibull and Inverse Weibull distributions and have extensively studied their properties. For example, Tojeiro *et al*. (2014) [15] introduced the complementary Weibull geometric distribution, while Afify *et al*. (2014) [16] developed the transmuted complementary Weibull geometric distribution. Then, Bera (2015) [17] proposed a novel distribution known as the Kumaraswamy Inverse Weibull Poisson (KIWP) distribution. Following that, Nofal *et al*. (2016) [18] developed the Kumaraswamy transmuted exponentiated additive Weibull distribution, and Abid and Mohammed (2016) [19] investigated the properties of distributions generated by mixing Weibull and Inverse Weibull distributions with zero-truncated Poisson. In the subsequent year, several new distributions such as the Marshall-Olkin additive Weibull and the Kumaraswamy complementary Weibull geometric distributions [20], and the Topp-Leone generated Weibull distribution [21] were introduced. Additionally, Vigas *et al*. (2017) [22] proposed a new long-term lifetime distribution with four parameters for risk competitive scenarios with decreasing, increasing, and unimodal hazard rate functions, which they named the Weibull-Poisson long-term distribution, while Okasha *et al*. (2017) [23] introduced the Extended Inverse Weibull distribution, focusing on its reliability applications. Following that, Cordeiro *et al*. (2018) [24] proposed the Lindley Weibull distribution, which accommodates unimodal, bathtub, and various monotone failure rates. Then, Basheer (2019) [25] and Al-Mualim (2019) [26] separately introduced the Alpha Power Inverse Weibull distribution and the extended Poisson Inverse Weibull distribution. Recently, Ahmad and Ghazal (2020) [27] presented the exponentiated additive Weibull distribution while Joshi and Kumar introduced the Lindley Inverse Weibull distribution [28] and the Poisson Inverse Weibull distributions [29]. Gbenga and Adeyemi (2022) [30] introduced a new four-parameter extended Inverse Weibull distribution known as the Alpha Power Extended Inverse Weibull Poisson distribution.

In this study, we focus on statistical inferences for the parameters of the Poisson Inverse Weibull (PIW) distribution developed by Joshi and Kumar (2021). The PIW distribution, a mixture of the Poisson and Inverse Weibull distributions, offers greater flexibility and a broader range of applicability compared to other lifetime distributions. Generally, the Poisson distribution is used to study the number of events occurring within a specified period or area, while the inverse Weibull distribution is employed to study the time until an event occurs. According to Joshi and Kumar (2021), the PIW distribution can be applied across various disciplines in a way that is similar to other lifetime distributions. While Joshi and Kumar [29] have made significant contributions by detailing the mathematical and statistical properties of the PIW distribution, including the probability density function, cumulative distribution function, survival function, hazard rate function, quantile function, skewness, and kurtosis, there remains a notable gap. Also, Joshi and Kumar [29] have proposed maximum likelihood estimators for the parameters of the PIW distribution, asymptotic confidence intervals, and the Fisher information matrix. However, research on the PIW distribution remains relatively limited, and there has been no comprehensive simulation study focused on statistical inferences related to its parameters, leaving a significant gap in the literature. Therefore, the main objective of this study is to introduce the signed log-likelihood ratio test (SLRT) for the scale parameter of the PIW distribution, which is crucial for assessing system failure rates. Additionally, this study evaluates the performance of the proposed method and compares it to an existing method introduced by Joshi and Kumar [29], which relies on the asymptotic normality of the maximum likelihood estimators.

## Poisson Inverse Weibull distribution

The Poisson Inverse Weibull (PIW) distribution is a novel lifetime distribution introduced by Joshi and Kumar (2021). It results from compounding the Poisson distribution and the Inverse Weibull (IW) distribution. This compounding procedure follows the methodology previously established by Adamidis and Loukas (1998) [31]. In this context, let *G*(*x*) and *g*(*x*) represent the baseline cumulative distribution function (CDF) and probability density function (PDF), respectively. The CDF of the Poisson family can be expressed as:

$$F(x;\lambda ,G(x))=\frac{1}{\left(1-\text{exp}(-\lambda )\right)}[1-\text{exp}(-\lambda G(x))],\;\;\;x>0,\lambda >0,$$ (1)

and its corresponding PDF is:

$$f(x;\lambda ,g(x),G(x))=\frac{1}{\left(1-\text{exp}(-\lambda )\right)}[\lambda g(x)\text{exp}(-\lambda G(x))],\;\;\;x>0,\lambda >0.$$ (2)

In this case, the Inverse Weibull distribution, with parameters ω (scale parameter) and β (shape parameter), is selected as the baseline distribution, with the following CDF and PDF:

$$G(x;\omega ,\beta )=\text{exp}(-\omega x^{-\beta }),\;\;\;x\geq 0,\omega >0,\beta >0,$$ (3)

and

$$g(x;\omega ,\beta )=\omega \beta x^{-(\beta +1)}\text{exp}(-\omega x^{-\beta }),\;\;\;x\geq 0,\omega >0,\beta >0.$$ (4)

By substituting the Eqs (3) and (4) into the Poisson family equations, the Poisson Inverse Weibull distribution is defined.

Let *X* be a non-negative random variable. The random variable *X* is considered to follow a PIW distribution if its CDF and PDF are defined as follows:

$$F(x;\omega ,\beta ,\lambda )=\frac{1}{\left(1-\text{exp}(-\lambda )\right)}\left[1-\text{exp}(-\lambda \text{exp}(-\omega x^{-\beta }))\right]$$ (5)

and

$$f(x;\omega ,\beta ,\lambda )=\frac{\omega \beta \lambda }{\left(1-\text{exp}(-\lambda )\right)}x^{-(1+\beta )}\text{exp}[-\lambda \text{exp}(-\omega x^{-\beta })-\omega x^{-\beta }],$$ (6)

where ω>0,β>0,λ>0 are scale, shape, and location parameters, respectively [29].

In this study, the PIW random variable (*X*) is generated using the following algorithm:

**Algorithm 1: Generating the PIW random variables.**

*Step 1:* Input the positive constants of the scale (ω), shape (β), and location (λ) parameters.

*Step 2:* Generate a uniform (0,1) random numbers, *w*.

*Step 3:* Generate a PIW random variable (*X*) using the following formula:

$$X=\omega {\left[-\text{ln}\left(-\frac{1}{\lambda }\text{ln}(1-(1-\text{exp}(-\lambda ))\omega )\right)\right]}^{-1/\beta }.$$ (7)

*Step 4:* Repeat *Step 2* through *3 n* times to obtain the PIW random variables of size *n* ($X_{1},X_{2},...,X_{n}$).

## Maximum likelihood estimator (MLE) of the parameters of the PIW distribution

In statistics, various methods are used to estimate population parameters. For point estimation, common techniques include the method of moments, maximum likelihood estimation, the method of least squares, and Bayesian estimation. This section focuses only on the maximum likelihood estimation, which is a widely used method with many good properties such as consistency, sufficiency, and efficiency. The following details explain how to obtain the maximum likelihood estimators for the parameters of the PIW distribution, including the formulation of the likelihood and log-likelihood functions, the steps for differentiating the log-likelihood function concerning the parameter of interest, and the techniques for solving the resulting equations.

Let $X_{1},X_{2},...,X_{n}$ be independent and identically distributed random variables from the PIW distribution. The likelihood function L(ω,β,λ|x) of the PIW, based on the probability density function in (6), is expressed as follows:

$$L(\omega ,\beta ,\lambda |x)={\left(\frac{\omega \beta \lambda }{1-\text{exp}(-\lambda )}\right)}^{n}\prod_{i=1}^{n}x_{i}^{-(1+\beta )}\text{exp}\left[\sum_{i=1}^{n}\left(-\lambda \text{exp}(-\omega x_{i}^{-\beta })-\omega x_{i}^{-\beta }\right)\right].$$ (8)

By taking the natural logarithm of the likelihood function in (8), we obtain the log-likelihood function l(ω,β,λ) as:

$$l(\omega ,\beta ,\lambda )=n\text{ln}(\omega \beta \lambda )-n\text{ln}(1-\text{exp}(-\lambda ))-(\beta +1)\sum_{i=1}^{n}\text{ln}(x_{i})-\omega \sum_{i=1}^{n}x_{i}^{-\beta }\;-\lambda \sum_{i=1}^{n}\text{exp}(-\omega x_{i}^{-\beta }).$$ (9)

To obtain the maximum likelihood estimates of the unknown parameters of the PIW distribution (ω,β,λ), we need to solve the following nonlinear equations by setting them equal to zero.

$$\frac{\partial l}{\partial \omega }=\frac{n}{\omega }-\sum_{i=1}^{n}x_{i}^{-\beta }+\lambda \sum_{i=1}^{n}x_{i}^{-\beta }\text{exp}(-\omega x_{i}^{-\beta })$$ (10)

$$\frac{\partial l}{\partial \beta }=\frac{n}{\beta }-\sum_{i=1}^{n}\text{ln}(x_{i})+\omega \sum_{i=1}^{n}x_{i}^{-\beta }\text{ln}(x_{i})-\omega \lambda \sum_{i=1}^{n}x_{i}^{-\beta }\text{ln}(x_{i})\text{exp}(-\omega x_{i}^{-\beta })$$ (11)

$$\frac{\partial l}{\partial \lambda }=\frac{n}{\lambda }-\frac{n}{\text{exp}(\lambda )-1}-\sum_{i=1}^{n}\text{exp}(-\omega x_{i}^{-\beta })$$ (12)

Regrettably, closed-form solutions for the maximum likelihood estimators (MLEs) of the parameters of the PIW distribution are not available. Additionally, proving the existence and uniqueness of these MLEs remains an unresolved issue. Therefore, iterative algorithms such as Newton-Raphson, Fisher scoring, and the expectation-maximization algorithm are required for finding the maximum likelihood estimates [32].

## Hypothesis testing for the scale parameter of the PIW distribution

The scale parameter is crucial in the statistical modeling of lifetime data because it provides valuable information for decision-making across various fields. For example, accurately estimating the scale parameter in maintenance planning helps predict system lifetimes, enabling better maintenance scheduling and reducing unexpected failures and downtime. In manufacturing, understanding the scale parameter ensures that products meet lifetime specifications and standards, maintaining consistent quality and performance. In critical industries, the scale parameter is essential for servicing or replacing components and systems before failure occurs, preventing accidents, and ensuring safety. In addition, accurate estimation of scale parameters helps companies optimize maintenance and replacement schedules, leading to cost savings by avoiding premature replacements and minimizing failure-related expenses. Therefore, this study focuses on hypothesis testing for the scale parameter (ω) of the PIW distribution, a crucial factor that influences the PDF and causes variations in the shape of the distribution.

The following subsections outline two hypothesis tests for ω, including an existing method that relies on the asymptotic normality of the MLEs and the proposed method that relies on the likelihood ratio test.

### Test based on the asymptotic normality of the maximum likelihood estimators (ANMLE)

The concept of using the asymptotic normality of the MLEs to infer the parameters of the PIW distribution was introduced by Joshi and Kumar (2021). This approach leverages the properties of MLEs when the sample size is large. It is important to note that the approach proposed by Joshi and Kumar (2021) is similar to the Wald method, which is based on the asymptotic distribution of MLEs for statistical inference.

Consider the problem of testing the scale parameter (ω). To perform a two-sided test for ω, the null (*H*_0_) and alternative (*H*_1_) hypothesis are stated as follows:


$$H_{0}:\omega =\omega_{0}$$

$$H_{1}:\omega \neq \omega_{0},$$ (13)

where $\omega_{0}$ represents the null value of the scale parameter ω of the PIW distribution.

Let θ=(ω,β,λ) be the parameter vector of the PIW distribution, and let $\hat{\theta }=(\hat{\omega },\hat{\beta },\hat{\lambda })$ be the corresponding MLE of θ. Then, under the large sample size, the asymptotic normality results in $(\hat{\theta }\;-\;\theta )\to N_{3}\left[0,(I(\theta )){\;}^{-1}\right]$, where I(θ) is the Fisher information matrix, and it is given by:

$$I(\theta )=-[\begin{array}{ccc} E\left(\frac{\partial^{2}l}{\partial \omega^{2}}\right) & E\left(\frac{\partial^{2}l}{\partial \omega \partial \beta }\right) & E\left(\frac{\partial^{2}l}{\partial \omega \partial \lambda }\right)\\ E\left(\frac{\partial^{2}l}{\partial \omega \partial \beta }\right) & E\left(\frac{\partial^{2}l}{\partial \beta^{2}}\right) & E\left(\frac{\partial^{2}l}{\partial \beta \partial \lambda }\right)\\ E\left(\frac{\partial^{2}l}{\partial \omega \partial \lambda }\right) & E\left(\frac{\partial^{2}l}{\partial \beta \partial \lambda }\right) & E\left(\frac{\partial^{2}l}{\partial \lambda^{2}}\right) \end{array}].$$ (14)

To clarify, we can further differentiate:

$$\frac{\partial^{2}l}{\partial \omega^{2}}=-\frac{n\beta }{\omega^{2}}-\lambda \sum_{i=1}^{n}x_{i}^{-2\beta }\text{exp}(-\omega x_{i}^{-\beta })$$ (15)

$$\frac{\partial^{2}l}{\partial \beta^{2}}=-\frac{n}{\beta^{2}}-\omega \sum_{i=1}^{n}x_{i}^{-\beta }[\text{ln}(x_{i}){]}^{2}-\omega^{2}\lambda \sum_{i=1}^{n}x_{i}^{-2\beta }[\text{ln}(x_{i}){]}^{2}\text{exp}(-\omega x_{i}^{-\beta })\;+\lambda \omega \sum_{i=1}^{n}x_{i}^{-\beta }[\text{ln}(x_{i}){]}^{2}\exp(-\omega x_{i}^{-\beta })$$ (16)

$$\frac{\partial^{2}l}{\partial \lambda^{2}}=-\frac{n}{\lambda^{2}}+\frac{n\text{exp}(\lambda )}{(\text{exp}(\lambda )-1{)}^{2}}$$ (17)

$$\frac{\partial^{2}l}{\partial \omega \partial \beta }=\sum_{i=1}^{n}x_{i}^{-\beta }\text{ln}(x_{i})+\lambda \omega \sum_{i=1}^{n}x_{i}^{-2\beta }\text{ln}(x_{i})\text{exp}(-\omega x_{i}^{-\beta })\;-\lambda \sum_{i=1}^{n}x_{i}^{-\beta }\text{ln}(x_{i})\text{exp}(-\omega x_{i}^{-\beta })$$ (18)

$$\frac{\partial^{2}l}{\partial \omega \partial \lambda }=\sum_{i=1}^{n}x_{i}^{-\beta }\text{exp}(-\omega x_{i}^{-\beta })$$ (19)

$$\frac{\partial^{2}l}{\partial \beta \partial \lambda }=-\omega \sum_{i=1}^{n}x_{i}^{-\beta }\text{ln}(x_{i})\text{exp}(-\omega x_{i}^{-\beta }).$$ (20)

In practice, the asymptotic variance of the MLE, represented as $[I(\theta ){]}^{-1}$, is not very useful because the value of θ is unknown. Therefore, the asymptotic variance can be approximated by substituting the estimated values of the parameters. A common procedure is to use the observed Fisher information matrix ($J(\hat{\theta })$) as an estimate of the true information matrix I(θ), and it can be obtained as

$$J(\hat{\theta })=-{\left[\begin{array}{ccc} \frac{\partial^{2}l}{\partial \omega^{2}} & \frac{\partial^{2}l}{\partial \omega \partial \beta } & \frac{\partial^{2}l}{\partial \omega \partial \lambda }\\ \frac{\partial^{2}l}{\partial \omega \partial \beta } & \frac{\partial^{2}l}{\partial \beta^{2}} & \frac{\partial^{2}l}{\partial \beta \partial \lambda }\\ \frac{\partial^{2}l}{\partial \omega \partial \lambda } & \frac{\partial^{2}l}{\partial \beta \partial \lambda } & \frac{\partial^{2}l}{\partial \lambda^{2}} \end{array}\right]}_{\left(\hat{\omega },\hat{\beta },\hat{\lambda }\right)}$$ (21)

where $\hat{\theta }=(\hat{\omega },\hat{\beta },\hat{\lambda })$ is the maximum likelihood estimator of θ=(ω,β,λ), and the variance-covariance matrix is then given by

$$[J(\hat{\theta }){]}^{-1}=\left[\begin{array}{ccc} Var(\hat{\omega }) & Cov(\hat{\omega },\hat{\beta }) & Cov(\hat{\omega },\hat{\lambda })\\ & \; & \;\\ Cov(\hat{\omega },\hat{\beta }) & Var(\hat{\beta }) & Cov(\hat{\beta },\hat{\lambda })\\ & \; & \;\\ Cov(\hat{\omega },\hat{\lambda }) & Cov(\hat{\beta },\hat{\lambda }) & Var(\hat{\lambda }) \end{array}\right].$$ (22)

Therefore, to test the hypothesis in (13), the test statistic based on the asymptotic normality of the MLEs for parameter ω is given by

$$Z(\omega )=\frac{\hat{\omega }-\omega_{0}}{\sqrt{Var(\hat{\omega })}},$$ (23)

where *H*_0_ is rejected at the significance level of α if $|Z(\omega )|\geq Z_{1-\alpha /2}$, and $Z_{1-\alpha /2}$ is the $100(1-\alpha /2){\;}^{th}$ percentile of the standard normal distribution.

### Signed log-likelihood ratio test (SLRT)

In statistics, the likelihood-ratio test (LRT) is a method used for hypothesis testing to infer the parameter of interest, proposed by Neyman and Pearson (1928) [33]. This test compares the goodness of fit between two competing statistical models. Typically, one model is derived by maximizing the likelihood over the entire parameter space, while the other model is obtained by imposing a constraint. If the observed data support the more constrained model (the null hypothesis), the likelihood of the two models should not differ significantly beyond what can be attributed to sampling error [34]. Thus, the likelihood-ratio test determines whether this ratio significantly differs from one or its natural logarithm significantly differs from zero.

The signed log-likelihood ratio test (SLRT), the proposed method, is an extension of the traditional LRT that incorporates a sign function to provide a more accurate inference, especially when dealing with small sample sizes or skewed distributions. In this section, we will begin by reviewing the traditional LRT method for testing the hypothesis stated in (13); this includes formulations of likelihood functions under the entire parameter space and the null hypothesis parameter space, the test statistic derived from this approach and its related distribution, and the criteria for concluding the test. Following this, we introduce the SLRT method, detailing its formulation, the associated distribution, and the criteria to draw conclusions based on the given test.

Consider the hypothesis in (13). Let Ω={(ω,β,λ):ω>0,β>0,λ>0} denotes the entire parameter space, and $\Omega_{0}=\{(\omega ,\beta ,\lambda ):\omega =\omega_{0},\beta =\beta_{\omega_{0}}>0,\lambda =\lambda_{\omega_{0}}>0\}$ denotes the null hypothesis parameter space. Then, the likelihood functions under the entire parameters space L(Ω) and the null hypothesis parameter space $L(\Omega_{0})$ can be obtained as follows:


L(Ω)=L(ω,β,λ)

$$={\left[\frac{\omega \beta \lambda }{1-\text{exp}(-\lambda )}\right]}^{n}\prod_{i=1}^{n}x_{i}^{-(1+\beta )}\text{exp}\left[\sum_{i=1}^{n}\left(-\lambda \text{exp}(-\omega x_{i}^{-\beta })-\omega x_{i}^{-\beta }\right)\right]$$ (24)

and


$$L(\Omega_{0})=L(\omega =\omega_{0},\beta_{\omega_{0}},\lambda_{\omega_{0}})$$

$$={\left[\frac{\omega_{0}\beta_{\omega_{0}}\lambda_{\omega_{0}}}{1-\text{exp}(-\lambda_{\omega_{0}})}\right]}^{n}\prod_{i=1}^{n}x_{i}^{-(1+\beta_{\omega_{0}})}\;\times \text{exp}\left[\sum_{i=1}^{n}\left(-\lambda_{\omega_{0}}\text{exp}(-\omega_{0}x_{i}^{-\beta_{\omega_{0}}})-\omega_{0}x_{i}^{-\beta_{\omega_{0}}}\right)\right],$$ (25)

respectively.

Let $L(\hat{\Omega })$ denotes the maximum of L(Ω) in Ω, and let $L({\hat{\Omega }}_{0})$ denotes the maximum of $L(\Omega_{0})$ in $\Omega_{0}$. Here, L(Ω) obtains its maximum value at $\hat{\omega },\hat{\beta },$ and $\hat{\lambda }$. Thus,


$$\text{max }L(\Omega )=L(\hat{\Omega })$$

$$={\left[\frac{\hat{\omega }\hat{\beta }\hat{\lambda }}{1-\text{exp}(-\hat{\lambda })}\right]}^{n}\prod_{i=1}^{n}x_{i}^{-(1+\hat{\beta })}\;\times \text{exp}\left[\sum_{i=1}^{n}\left(-\hat{\lambda }\text{exp}(-\hat{\omega }x_{i}^{-\hat{\beta }})-\hat{\omega }x_{i}^{-\hat{\beta }}\right)\right],$$ (26)

where $\left(\hat{\omega },\hat{\beta },\hat{\lambda }\right)$ denotes the maximum likelihood estimators of (ω,β,λ), and we obtain the maximum of $L(\Omega_{0})$ as follows:


$$\text{max }L(\Omega_{0})=L({\hat{\Omega }}_{0})$$

$$={\left[\frac{\omega_{0}{\hat{\beta }}_{\omega_{0}}{\hat{\lambda }}_{\omega_{0}}}{1-\text{exp}(-{\hat{\lambda }}_{\omega_{0}})}\right]}^{n}\prod_{i=1}^{n}x_{i}^{-(1+{\hat{\beta }}_{\omega_{0}})}\;\times \text{exp}\left[\sum_{i=1}^{n}\left(-{\hat{\lambda }}_{\omega_{0}}\text{exp}(-\omega_{0}x_{i}^{-{\hat{\beta }}_{\omega_{0}}})-\omega_{0}x_{i}^{-{\hat{\beta }}_{\omega_{0}}}\right)\right],$$ (27)

where $\left({\hat{\beta }}_{\omega_{0}},{\hat{\lambda }}_{\omega_{0}}\right)$ denotes the constrained maximum likelihood estimator of $\left(\beta_{\omega_{0}},\lambda_{\omega_{0}}\right)$ for a fixed $\omega =\omega_{0}$. Then, we base the hypothesis test in (13) on the following statistic:

Q=−2lnΛ, (28)

where Λ is the likelihood ratio, and it is defined as $\Lambda =\frac{L({\hat{\Omega }}_{0})}{L(\hat{\Omega })}$.

Under certain regularity conditions (see S1 Appendix) and *H*_0_, the result of Wilk’s theorem verifies that the distribution of the test statistic Q=−2lnΛ converges in distribution (as *n*→∞) to a chi-squared distribution with *p* degrees of freedom; this can be expressed as:

$$-2\text{ln}\Lambda \overset{d}{\to }\chi_{p}^{2},$$ (29)

where *p* is equal to the difference in the dimensionality of the full parameter space and the subset of the parameter space associated with *H*_0_. In our case, *p* is equal to 1. Therefore, if ${\text{c}}^{*}$ satisfies $P(\chi_{p}^{2}\geq c^{*})=\alpha$, then the rejection region $R=\{x:-2\text{ln}\Lambda \geq c^{*}\}$ gives an approximate size α test for large sample sizes, and *H*_0_ is rejected if $-2\text{ln}\Lambda \geq {\chi^{2}}_{1,\alpha }$ [35].

Using the statistic from Eq (28), we can easily derive the SLRT statistic. As a result, the SLRT statistic can be obtained as [36]:

$$R(\omega )=sign(\hat{\omega }-\omega_{0})\sqrt{Q},$$ (30)

where $sign(\hat{\omega }-\omega_{0})=1$ if $(\hat{\omega }-\omega_{0})>0$, and $sign(\hat{\omega }-\omega_{0})=-1$ if $(\hat{\omega }-\omega_{0})<0$.

Under *H*_0_, it is known that R(ω) is approximately distributed as a standard normal with first-order accuracy [37–40]. Therefore, *H*_0_ is rejected at the significance level of α if $|R(\omega )|\geq Z_{1-\alpha /2}$, where $Z_{1-\alpha /2}$ is the $100(1-\alpha /2){\;}^{th}$ percentile of the standard normal distribution.

**Fig 1:**
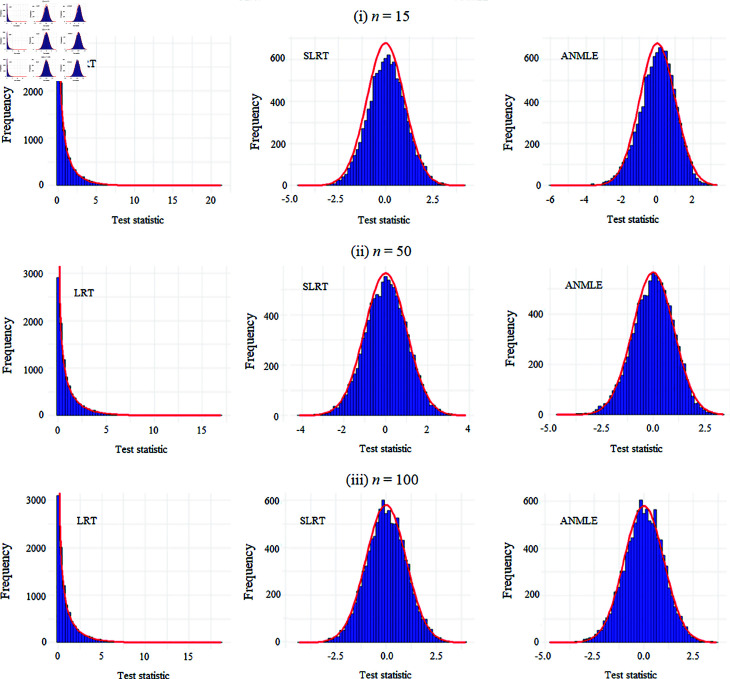
Distributions of test statistics under simulated conditions. Histograms of 10,000 simulated values of the LRT, SLRT, and ANMLE statistics for sample sizes *n* = 15, 50, 100, generated under the parameter setting (ω,β,λ)=(1.25,1,3.5). Red reference lines indicate the corresponding theoretical distributions.

Fig 1 displays histograms of 10000 simulated values for three statistical methods: the likelihood ratio test (LRT), signed log-likelihood ratio test (SLRT), and asymptotic normality of the MLEs (ANMLE), across different sample sizes (*n* = 15, 50, 100). The LRT statistic shows a right-skewed distribution corresponding to its theoretical distribution (chi-square), represented by the red line. In contrast, the SLRT and ANMLE statistics closely follow a standard normal distribution, as evidenced by their bell-shaped histograms and alignment with the theoretical distribution (red line). The agreement between the distributions of the SLRT and ANMLE statistics and the theoretical distribution improves as the sample size (*n*) increases, demonstrating their better asymptotic properties.

## Methods of evaluating hypothesis tests

When deciding whether to accept or reject the null hypothesis (*H*_0_), a researcher may make an error. Typically, the performance of hypothesis tests is assessed based on their probabilities of making errors. In this section, we will discuss these error probabilities in detail.

### Probability of type I error

A type I error occurs when the null hypothesis is rejected while it is actually true. The probability of making this type of error is denoted by α, which is also known as the significance level of the test.

In this study, we evaluate the efficiency of the proposed tests using the empirical type I error rate ($\hat{\alpha }$). This rate is determined by dividing the number of times the null hypothesis is rejected when true by the number of replications. Essentially, it measures how often the test incorrectly identifies an effect when there is none. According to Cochran’s criterion [41], a test is considered effective in controlling the probability of a type I error if the empirical type I error rate falls within the interval [0.04, 0.06] at the significance level of 0.05. This criterion ensures that the test maintains a balance between sensitivity and specificity, thereby minimizing the chances of false positives [42].

### Probability of type II error and power of a test

A Type II error occurs when the null hypothesis is incorrectly accepted as true, despite it being false. The probability of making this type of error is represented by β.

To evaluate the performance of a statistical test, we use the concept of power, which is defined as 1−β. The power of a test is the probability of correctly rejecting the null hypothesis when it is indeed false. The empirical power ($1\;-\;\hat{\beta }$) can be calculated by dividing the number of times the null hypothesis is rejected (when it is false) by the total number of trials. Essentially, a test with higher power is considered more efficient because it has a greater ability to detect true effects.

## Simulation study

In this study, we aim to propose a likelihood-based method for testing the scale parameter of the PIW distribution and compare its performance to the existing method through a comprehensive Monte Carlo simulation. Specifically, we focus on comparing the empirical type I error rates and powers [43–45] of the signed log-likelihood ratio test (SLRT) and the test based on the asymptotic normality of the MLEs (ANMLE). By conducting 10,000 replications, we ensure a robust assessment of these methods under different conditions.

**Fig 2:**
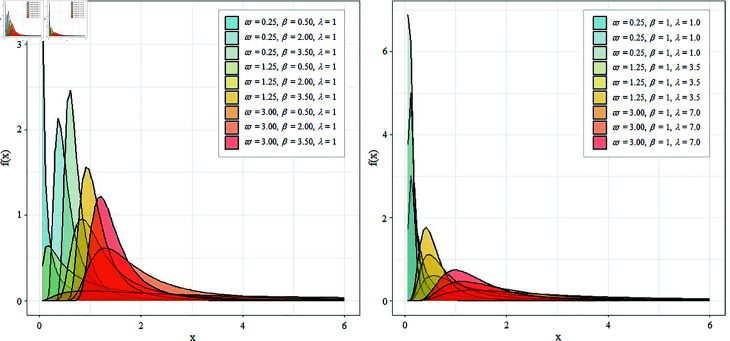
Probability density function of the PIW distribution. The figure shows the probability density function (PDF) of the PIW distribution for various combinations of the parameters ω,β, and λ.

As mentioned earlier, this study focuses on conducting a two-sided test for the scale parameter (ω), which influences the peakedness and tail behavior of the PIW distribution (see Fig 2). A low-scale parameter results in a probability density function (PDF) that is highly right-skewed, with a steep peak near zero and a long, heavy tail, indicating a highly dispersed data spread with high probability density near lower values of data points. In contrast, a high-scale parameter leads to a more flattened PDF, and the peak shifts further to the right, showing that the probability density is more evenly spread, with a broader peak and a smoother decline in the tail [46]. To ensure that our simulation study captures a broad range of realistic scenarios, we selected representative values for the scale parameter, ranging from 0.1 to 80. These values were chosen based on theoretical considerations of the shape of the PIW distribution, as well as from prior empirical studies [29]. Additionally, we consider the location parameter (λ) at values of 1, 3.5, and 7 while keeping the shape parameter (β) fixed at 1. We also explore scenarios where the shape parameter (β) is set to 0.2, 2, and 3.5, with the location parameter (λ) fixed at 1. To account for different sample sizes (*n*), we define small samples as 10 and 15, medium samples as 30 and 50, and large samples as 80 and 100. This experimental setup enables us to observe how the scale, shape, and location parameters, as well as sample sizes, influence the type I error rate and the power.

### Simulation procedure

To study the probability of type I error and the power of the test, the following algorithms are used to evaluate and compare the performance of the proposed methods.

**Algorithm 2: Calculating the empirical type I error rate.**

*Step 1:* Define the hypothesis of the test as follows:


$$H_{0}:\omega =\omega_{0}\;\text{versus}\;H_{1}:\omega \neq \omega_{0}.$$

*Step 2:* Generate the PIW data using Algorithm 1, assuming the null hypothesis (*H*_0_) from *Step 1* is true, and under the specified parameters scope.

*Step 3:* Compute the test statistics for the SLRT and ANMLE methods using Eqs (23) and (30), respectively.

*Step 4:* Evaluate whether the null hypothesis $H_{0}:\omega =\omega_{0}$ using statistics in *Step 3* is rejected at the significance level (α) of 0.05.

*Step 5:* Repeat *Step 2* through *4* a total of 10000 times for each situation.

*Step 6:* Calculate the empirical type I error rate ($\hat{\alpha }$) using the following formula:


$$\hat{\alpha }=\frac{\text{Number of times}\;H_{0}:\omega =\omega_{0}\;\text{is rejected when true}}{10,000}.$$

**Algorithm 3: Calculating the empirical power.**

*Step 1:* Define the hypothesis of the test as follows:


$$H_{0}:\omega =\omega_{0}\;\text{versus}\;H_{1}:\omega =\omega_{1}.$$

*Step 2:* Generate the PIW data using Algorithm 1, assuming the alternative hypothesis (*H*_1_) from *Step 1* is true, and under the specified parameters scope.

*Step 3:* Compute the test statistics for the SLRT and ANMLE methods using Eqs (23) and (30), respectively.

*Step 4:* Evaluate whether the null hypothesis $H_{0}:\omega =\omega_{0}$ using statistics in *Step 3* is rejected at the significance level (α) of 0.05.

*Step 5:* Repeat *Step 2* through *4* a total of 10000 times for each situation.

*Step 6:* Calculate the empirical power ($1-\hat{\beta }$) using the following formula:


$$1-\hat{\beta }=\frac{\text{Number of times}\;H_{0}:\omega =\omega_{0}\;\text{is rejected when}\;H_{1}:\omega =\omega_{1}\;\text{is true}}{10,000}.$$

### Simulation results

The simulation results for the empirical type I error rates and the powers of the proposed methods are presented in Tables 1–8.

**Table 1:** Empirical type I error rates for testing $H_{0}:\omega =\omega_{0}$ versus $H_{1}:\omega \neq \omega_{0}$ when β=1.

n	Method	λ=1			λ=3.5			λ=7		
		0.25	1.25	3	0.25	1.25	$\omega_{0}=3$	0.25	1.25	3
10	SLRT	0.0499	0.0542	0.0500	0.0521	0.0536	0.0532	0.0576	0.0561	0.0517
	ANMLE	0.0450	0.0524	0.0525	0.0464	0.0494	0.0503	0.0454	0.0539	0.0524
15	SLRT	0.0537	0.0513	0.0466	0.0532	0.0512	0.0541	0.0499	0.0548	0.0519
	ANMLE	0.0475	0.0508	0.0472	0.0500	0.0465	0.0547	0.0454	0.0504	0.0503
30	SLRT	0.0496	0.0501	0.0485	0.0506	0.0517	0.0469	0.0495	0.0492	0.0511
	ANMLE	0.0460	0.0494	0.0508	0.0499	0.0500	0.0466	0.0485	0.0495	0.0525
50	SLRT	0.0489	0.0519	0.0536	0.0521	0.0468	0.0492	0.0508	0.0475	0.0486
	ANMLE	0.0468	0.0499	0.0533	0.0516	0.0475	0.0508	0.0477	0.0471	0.0499
80	SLRT	0.0502	0.0499	0.0521	0.0518	0.0542	0.0473	0.0502	0.0519	0.0480
	ANMLE	0.0481	0.0467	0.0526	0.0532	0.0515	0.0478	0.0497	0.0521	0.0501
100	SLRT	0.0486	0.0512	0.0510	0.0504	0.0508	0.0507	0.0535	0.0474	0.0510
	ANMLE	0.0482	0.0519	0.0513	0.0501	0.0499	0.0494	0.0541	0.0468	0.0504

**Table 2:** Empirical type I error rates for testing $H_{0}:\omega =\omega_{0}$ versus $H_{1}:\omega \neq \omega_{0}$ when λ=1.

n	Method	β=0.5			β=2			β=3.5		
		0.25	1.25	3	0.25	1.25	3	0.25	1.25	3
10	SLRT	0.0510	0.0479	0.0543	0.0535	0.0488	0.0517	0.0534	0.0556	0.0509
	ANMLE	0.0433	0.0459	0.0503	0.0459	0.0445	0.0468	0.0491	0.0458	0.0458
15	SLRT	0.0503	0.0533	0.0533	0.0539	0.0516	0.0503	0.0551	0.0512	0.0504
	ANMLE	0.0472	0.0482	0.0475	0.0472	0.0466	0.0466	0.0530	0.0463	0.0481
30	SLRT	0.0466	0.0519	0.0521	0.0484	0.0548	0.0506	0.0486	0.0509	0.0501
	ANMLE	0.0431	0.0500	0.0488	0.0454	0.0542	0.0480	0.0475	0.0523	0.0489
50	SLRT	0.0529	0.0532	0.0473	0.0509	0.0496	0.0511	0.0525	0.0505	0.0527
	ANMLE	0.0526	0.0525	0.0474	0.0495	0.0466	0.0501	0.0515	0.0494	0.0516
80	SLRT	0.0491	0.0461	0.0504	0.0501	0.0519	0.0524	0.0490	0.0481	0.0546
	ANMLE	0.0485	0.0444	0.0499	0.0500	0.0511	0.0488	0.0477	0.0496	0.0539
100	SLRT	0.0464	0.0518	0.0535	0.0500	0.0478	0.0483	0.0451	0.0528	0.0538
	ANMLE	0.0475	0.0503	0.0550	0.0491	0.0473	0.0489	0.0446	0.0521	0.0515

**Table 3:** Empirical powers for testing $H_{0}:\omega =0.25$ versus $H_{1}:\omega =\omega_{1}$ when β=1.

n	Method	λ=1			λ=3.5			λ=7		
		$\omega_{1}=0.3$	$\omega_{1}=0.4$	$\omega_{1}=0.5$	$\omega_{1}=0.3$	$\omega_{1}=0.4$	$\omega_{1}=0.5$	$\omega_{1}=0.3$	$\omega_{1}=0.4$	$\omega_{1}=0.5$
10	SLRT	0.0998	0.3552	0.6699	0.1386	0.8758	0.9205	0.2023	0.8411	0.9929
	ANMLE	0.039	0.1709	0.4195	0.0788	0.6752	0.8386	0.1354	0.7545	0.9853
15	SLRT	0.1205	0.5169	0.8627	0.1799	0.9775	0.9885	0.2808	0.9548	0.9998
	ANMLE	0.0605	0.3395	0.7221	0.1191	0.9279	0.9732	0.2088	0.9260	0.9997
30	SLRT	0.1942	0.8262	0.9933	0.3209	0.9998	0.9999	0.5042	0.9994	1.0000
	ANMLE	0.1346	0.7482	0.9864	0.2595	0.9997	0.9999	0.4416	0.9989	1.0000
50	SLRT	0.2976	0.9663	0.9999	0.4959	1.0000	1.0000	0.7227	1.0000	1.0000
	ANMLE	0.2378	0.9468	0.9997	0.4415	1.0000	1.0000	0.6822	1.0000	1.0000
80	SLRT	0.4447	0.9983	1.0000	0.6951	1.0000	1.0000	0.9007	1.0000	1.0000
	ANMLE	0.3882	0.9971	1.0000	0.6584	1.0000	1.0000	0.8841	1.0000	1.0000
100	SLRT	0.5303	0.9998	1.0000	0.7965	1.0000	1.0000	0.9531	1.0000	1.0000
	ANMLE	0.4787	0.9996	1.0000	0.7677	1.0000	1.0000	0.9427	1.0000	1.0000

**Table 4:** Empirical powers for testing $H_{0}:\omega =1.25$ versus $H_{1}:\omega =\omega_{1}$ when β=1.

n	Method	λ=1			λ=3.5			λ=7		
		$\omega_{1}=1.4$	$\omega_{1}=1.5$	$\omega_{1}=1.6$	$\omega_{1}=1.4$	$\omega_{1}=1.5$	$\omega_{1}=1.6$	$\omega_{1}=1.4$	$\omega_{1}=1.5$	$\omega_{1}=1.6$
10	SLRT	0.0735	0.0998	0.1371	0.0879	0.1386	0.2113	0.1109	0.2023	0.3273
	ANMLE	0.0322	0.0390	0.0540	0.0489	0.0788	0.1253	0.0693	0.1354	0.2320
15	SLRT	0.0775	0.1205	0.1795	0.0999	0.1799	0.2942	0.1372	0.2808	0.4755
	ANMLE	0.0423	0.0605	0.0928	0.0655	0.1191	0.2058	0.0964	0.2088	0.3811
30	SLRT	0.1058	0.1942	0.3176	0.1547	0.3209	0.5285	0.2279	0.5042	0.7659
	ANMLE	0.0691	0.1346	0.2297	0.1174	0.2595	0.4511	0.1867	0.4416	0.7195
50	SLRT	0.1428	0.2976	0.4952	0.2279	0.4959	0.7578	0.3525	0.7227	0.9344
	ANMLE	0.1075	0.2378	0.4183	0.1886	0.4415	0.7074	0.3118	0.6822	0.9186
80	SLRT	0.2027	0.4447	0.6963	0.3313	0.6951	0.9258	0.5143	0.9007	0.9930
	ANMLE	0.1672	0.3882	0.6404	0.2939	0.6584	0.9078	0.4779	0.8841	0.9909
100	SLRT	0.2421	0.5303	0.7986	0.4028	0.7965	0.9665	0.6114	0.9531	0.9985
	ANMLE	0.2052	0.4787	0.7585	0.3703	0.7677	0.9576	0.5795	0.9427	0.9980

**Table 5:** Empirical powers for testing $H_{0}:\omega =3$ versus $H_{1}:\omega =\omega_{1}$ when β=1.

n	Method	λ=1			λ=3.5			λ=7		
		$\omega_{1}=3.3$	$\omega_{1}=3.5$	$\omega_{1}=3.7$	$\omega_{1}=3.3$	$\omega_{1}=3.5$	$\omega_{1}=3$	$\omega_{1}=3.3$	$\omega_{1}=3.5$	$\omega_{1}=3.7$
10	SLRT	0.0686	0.0879	0.1159	0.0776	0.1154	0.1687	0.0930	0.1597	0.2507
	ANMLE	0.0314	0.0355	0.0429	0.0453	0.0649	0.0973	0.0595	0.1038	0.1724
15	SLRT	0.0696	0.1003	0.1416	0.0865	0.1424	0.2253	0.1105	0.2122	0.3587
	ANMLE	0.0397	0.0499	0.0735	0.0568	0.0928	0.1496	0.0785	0.1558	0.2741
30	SLRT	0.0880	0.1548	0.2410	0.1257	0.2442	0.4041	0.1758	0.3806	0.6227
	ANMLE	0.0577	0.1025	0.1703	0.0926	0.1916	0.335	0.1429	0.3216	0.5624
50	SLRT	0.1159	0.2280	0.3789	0.1750	0.3771	0.6127	0.2642	0.5781	0.8377
	ANMLE	0.0867	0.1749	0.3071	0.1410	0.3259	0.5618	0.2280	0.5322	0.8086
80	SLRT	0.1572	0.3341	0.5506	0.2491	0.5457	0.8161	0.3877	0.7805	0.9636
	ANMLE	0.1276	0.2847	0.4950	0.2203	0.5034	0.7863	0.3545	0.7521	0.9568
100	SLRT	0.1850	0.4058	0.6581	0.3069	0.6524	0.8948	0.4668	0.8640	0.9863
	ANMLE	0.1542	0.3613	0.6055	0.2698	0.6159	0.8749	0.4367	0.8457	0.9835

**Table 6:** Empirical powers for testing $H_{0}:\omega =0.25$ versus $H_{1}:\omega =\omega_{1}$ when λ=1.

n	Method	β=0.5			β=2			β=3.5		
		$\omega_{1}=0.3$	$\omega_{1}=0.4$	$\omega_{1}=0.5$	$\omega_{1}=0.3$	$\omega_{1}=0.4$	$\omega_{1}=0.5$	$\omega_{1}=0.3$	$\omega_{1}=0.4$	$\omega_{1}=0.5$
10	SLRT	0.0904	0.3648	0.6621	0.0954	0.3557	0.6684	0.0999	0.3588	0.6715
	ANMLE	0.0332	0.1755	0.4181	0.0365	0.1664	0.4178	0.0379	0.1711	0.4280
15	SLRT	0.1247	0.5159	0.8529	0.1188	0.5099	0.8489	0.1196	0.5084	0.8536
	ANMLE	0.0615	0.3419	0.7210	0.0587	0.3413	0.7120	0.0600	0.3275	0.7135
30	SLRT	0.1968	0.8197	0.9942	0.1936	0.8315	0.9933	0.2000	0.8233	0.9927
	ANMLE	0.1336	0.7422	0.9862	0.1355	0.7513	0.9854	0.1391	0.7391	0.9842
50	SLRT	0.3015	0.9700	1.0000	0.3045	0.9679	1.0000	0.3038	0.9704	1.0000
	ANMLE	0.2403	0.9491	0.9997	0.2441	0.9502	0.9999	0.2371	0.9505	0.9999
80	SLRT	0.4454	0.9984	1.0000	0.4488	0.9984	1.0000	0.4489	0.9982	1.0000
	ANMLE	0.3897	0.9972	1.0000	0.3939	0.9969	1.0000	0.3957	0.9975	1.0000
100	SLRT	0.5380	1.0000	1.0000	0.5306	0.9998	1.0000	0.5367	0.9999	1.0000
	ANMLE	0.4870	0.9999	1.0000	0.4812	0.9997	1.0000	0.4866	0.9998	1.0000

**Table 7:** Empirical powers for testing $H_{0}:\omega =1.25$ versus $H_{1}:\omega =\omega_{1}$ when λ=1.

n	Method	β=0.5			β=2			β=3.5		
		$\omega_{1}=1.4$	$\omega_{1}=1.5$	$\omega_{1}=1.6$	$\omega_{1}=1.4$	$\omega_{1}=1.5$	$\omega_{1}=1.6$	$\omega_{1}=1.4$	$\omega_{1}=1.5$	$\omega_{1}=1.6$
10	SLRT	0.0684	0.0976	0.1403	0.0718	0.0942	0.1402	0.0630	0.1019	0.1445
	ANMLE	0.0309	0.0364	0.0533	0.0300	0.0344	0.0534	0.0300	0.0418	0.0554
15	SLRT	0.0766	0.1151	0.1842	0.0819	0.1173	0.1809	0.0750	0.1266	0.1785
	ANMLE	0.0385	0.0583	0.0901	0.0453	0.0599	0.0954	0.0394	0.0650	0.0959
30	SLRT	0.1091	0.1916	0.3160	0.1085	0.1959	0.3104	0.1033	0.1937	0.3281
	ANMLE	0.0718	0.1278	0.2265	0.0731	0.1333	0.2295	0.0681	0.1318	0.2406
50	SLRT	0.1398	0.2895	0.4940	0.1449	0.3088	0.4946	0.1431	0.2966	0.4899
	ANMLE	0.1037	0.2322	0.4199	0.1072	0.2462	0.4184	0.1074	0.2345	0.4155
80	SLRT	0.2040	0.4346	0.7051	0.2103	0.4493	0.7042	0.2074	0.4490	0.7044
	ANMLE	0.1679	0.3780	0.6507	0.1709	0.3932	0.6531	0.1702	0.3900	0.6520
100	SLRT	0.2413	0.5293	0.7970	0.2444	0.5353	0.8019	0.2444	0.5383	0.7941
	ANMLE	0.2066	0.4763	0.7596	0.2078	0.4815	0.7650	0.2085	0.4855	0.7561

**Table 8:** Empirical powers for testing $H_{0}:\omega =3$ versus $H_{1}:\omega =\omega_{1}$ when λ=1.

n	Method	β=0.5			β=2			β=3.5		
		$\omega_{1}=3.3$	$\omega_{1}=3.5$	$\omega_{1}=3.7$	$\omega_{1}=3.3$	$\omega_{1}=3.5$	$\omega_{1}=3.7$	$\omega_{1}=3.3$	$\omega_{1}=3.5$	$\omega_{1}=3.7$
10	SLRT	0.0654	0.0857	0.1122	0.0689	0.0858	0.1071	0.0640	0.0861	0.1106
	ANMLE	0.0323	0.0336	0.0426	0.0329	0.0352	0.0388	0.0322	0.0338	0.0419
15	SLRT	0.0718	0.1058	0.1467	0.0711	0.0985	0.1510	0.0703	0.0989	0.1479
	ANMLE	0.0430	0.0552	0.0740	0.0382	0.0501	0.0740	0.0436	0.0531	0.0738
30	SLRT	0.0899	0.1514	0.2416	0.0884	0.1539	0.2499	0.0900	0.1547	0.2393
	ANMLE	0.0595	0.1003	0.1703	0.0597	0.1009	0.1756	0.0594	0.1052	0.1681
50	SLRT	0.1182	0.2327	0.3851	0.1205	0.2332	0.3784	0.1115	0.2263	0.3786
	ANMLE	0.0879	0.1813	0.3140	0.0895	0.1776	0.3070	0.0822	0.1753	0.3104
80	SLRT	0.1561	0.3410	0.5529	0.1565	0.3365	0.5525	0.1615	0.3344	0.5562
	ANMLE	0.1240	0.2866	0.4939	0.1246	0.2886	0.4942	0.1318	0.2822	0.4973
100	SLRT	0.1949	0.4003	0.6672	0.1910	0.4130	0.6515	0.1888	0.4093	0.6450
	ANMLE	0.1636	0.3507	0.6156	0.1593	0.3635	0.6036	0.1569	0.3611	0.5920

**Fig 3:**
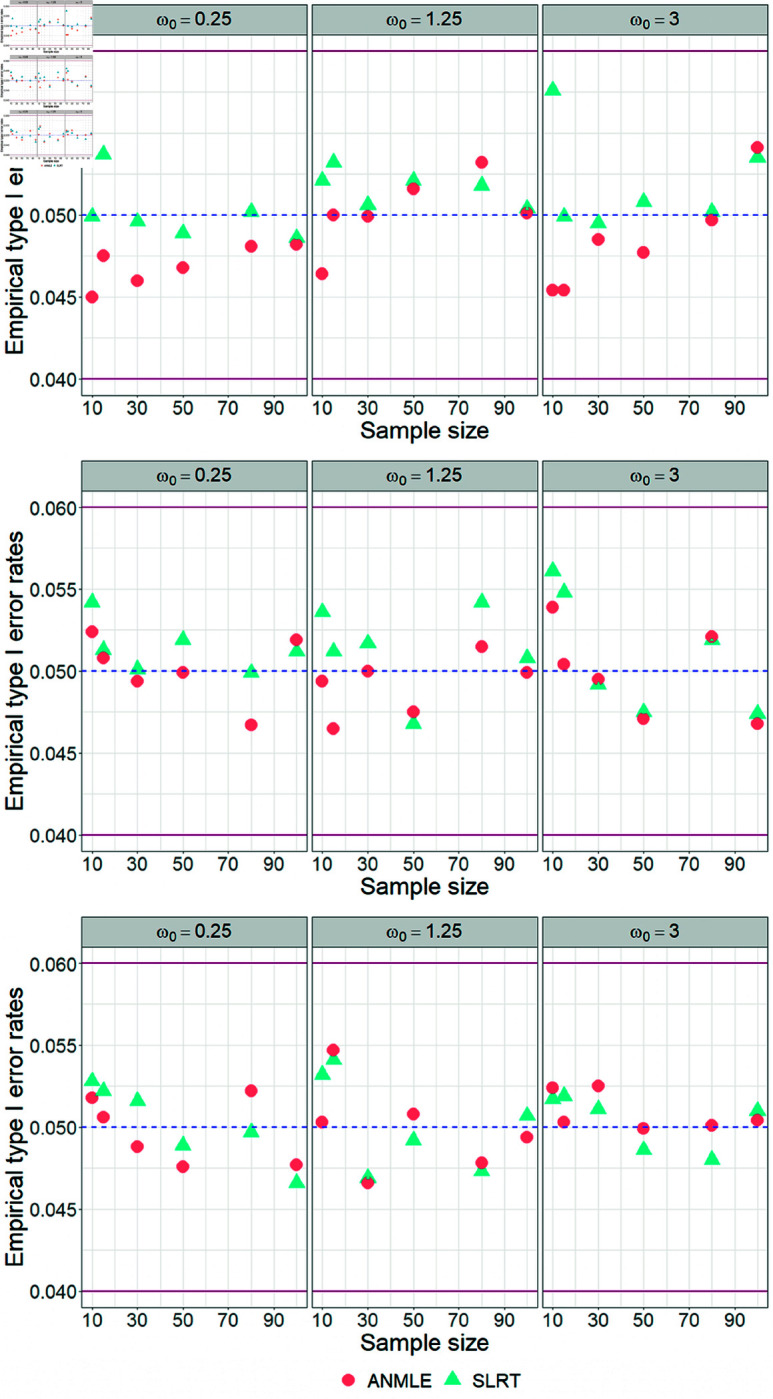
Empirical type I error rates under the null hypothesis $H_{0}:\omega =\omega_{0}$ with a fixed β. The figure shows the ability of SLRT and ANMLE methods to control the type I error rate when β is fixed as 1 and λ is set to 1, 3.5, and 7. Results are presented for varying values of $\omega_{0}$ and sample size (*n*), under the alternative hypothesis $H_{1}:\omega \neq \omega_{0}$.

**Fig 4:**
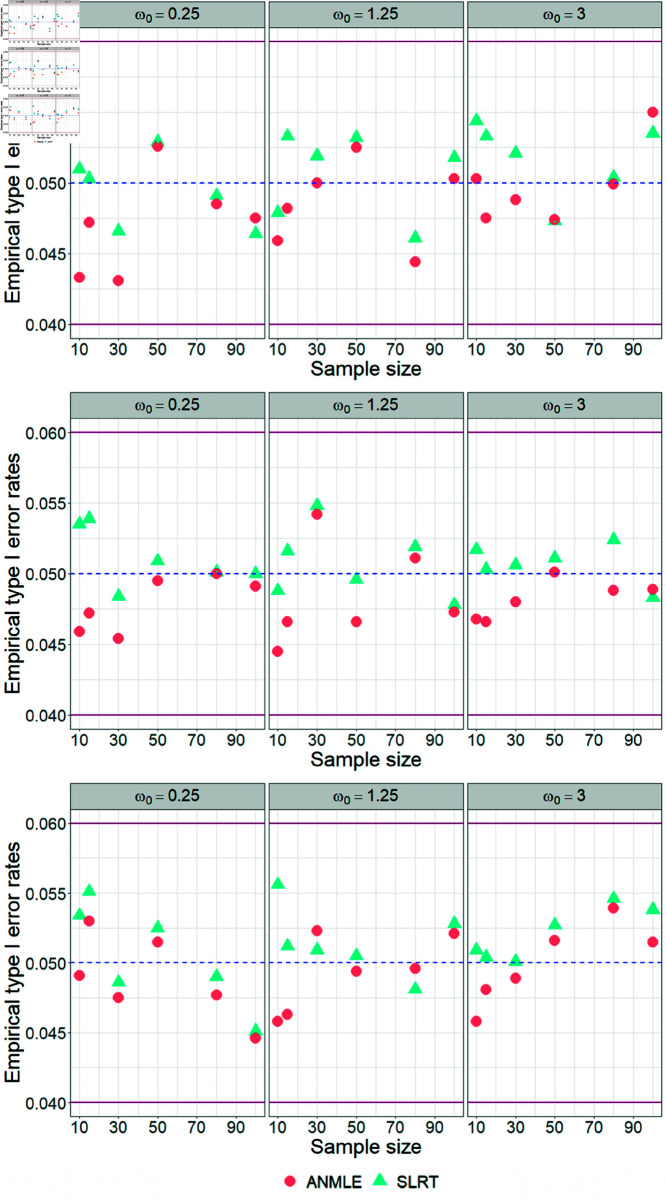
Empirical type I error rates under the null hypothesis $H_{0}:\omega =\omega_{0}$ with a fixed λ. The figure shows the ability of SLRT and ANMLE methods to control the type I error rate when λ is fixed as 1 and β is set to 0.5, 2, and 3.5. Results are presented for varying values of $\omega_{0}$ and sample size (*n*), under the alternative hypothesis $H_{1}:\omega \neq \omega_{0}$.

Regarding Tables 1 and 2, it can be found that both the SLRT and the ANMLE methods effectively control the probability of type I errors across all scenarios at the significance level (α) 0.05. This conclusion is demonstrated by the empirical type I error rates of both methods, which fall within Cochran’s criterion [41] (ranging from 0.04 to 0.06), as illustrated in Figs 3 and 4. Moreover, we also observed that the ANMLE method tends to be conservative, as its empirical type I error rates are often lower than the specified significance level (α), especially when λ=1.

For the power study, we are interested in testing $H_{0}:\omega =\omega_{0}$ versus $H_{1}:\omega =\omega_{1}$ at the significance level (α) of 0.05, and the simulation results are reported in Tables 3–8.

**Fig 5:**
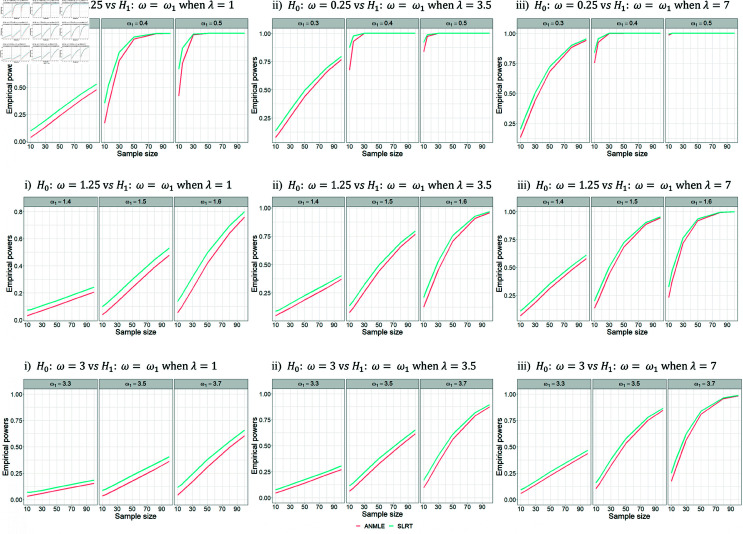
Empirical powers for testing $H_{0}:\omega =\omega_{0}$ versus $H_{1}:\omega =\omega_{1}$ with a fixed β. The figure shows the empirical powers of SLRT and ANMLE methods when β is fixed as 1 and λ is set to 1, 3.5, and 7. Results are presented for varying values of $\omega_{0}$, $\omega_{1}$, and sample size (*n*).

According to Tables 3–5, we set the parameter λ to 1, 3.5, and 7 while keeping the parameter β fixed at 1. The simulation results indicate that the SLRT demonstrates higher empirical power than the ANMLE, even with small sample sizes (n=10,15). In cases of testing the hypothesis $H_{0}:\omega =0.25$ versus $H_{1}:\omega =\omega_{1}$ with medium (n=30,50) and large (n=80,100) sample sizes, it has been found that the power of the ANMLE approaches that of the SLRT test as the ratio $\omega_{0}/\omega_{1}$ increases. However, there are no instances where the ANMLE outperforms the SLRT. Furthermore, we also observe that the SLRT and ANMLE generally exhibit the expected power behavior; their empirical powers tend to increase with larger sample sizes and higher ratios of $\omega_{0}/\omega_{1}$ (see Fig 5).

**Fig 6:**
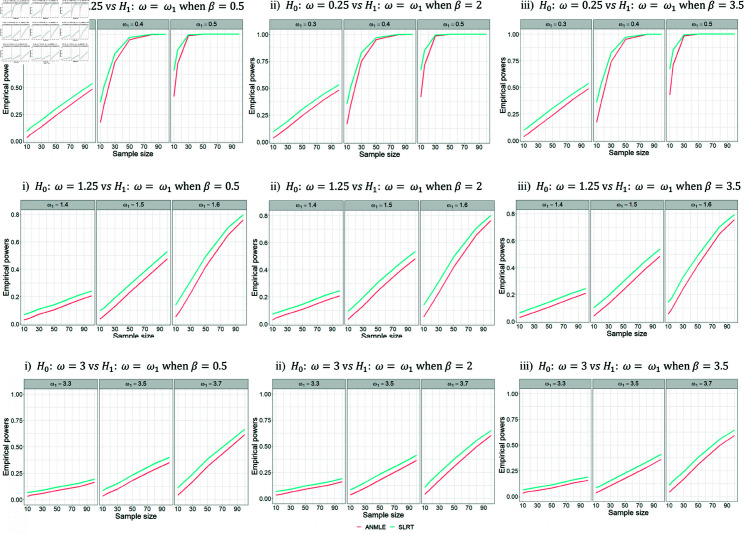
Empirical powers for testing $H_{0}:\omega =\omega_{0}$ versus $H_{1}:\omega =\omega_{1}$ with a fixed λ. The figure shows the empirical powers of SLRT and ANMLE methods when λ is fixed as 1 and β is set to 0.5, 2, and 3.5. Results are presented for varying values of $\omega_{0}$, $\omega_{1}$, and sample size (*n*).

In contrast, in Tables 6–8, we set the parameter β to values of 0.5, 2, and 3.5 while keeping the parameter λ fixed at 1. The simulation results showed that the empirical powers across all cases were nearly identical, as shown in Fig 6, indicating that the value of β did not affect the power of the test.

**Fig 7:**
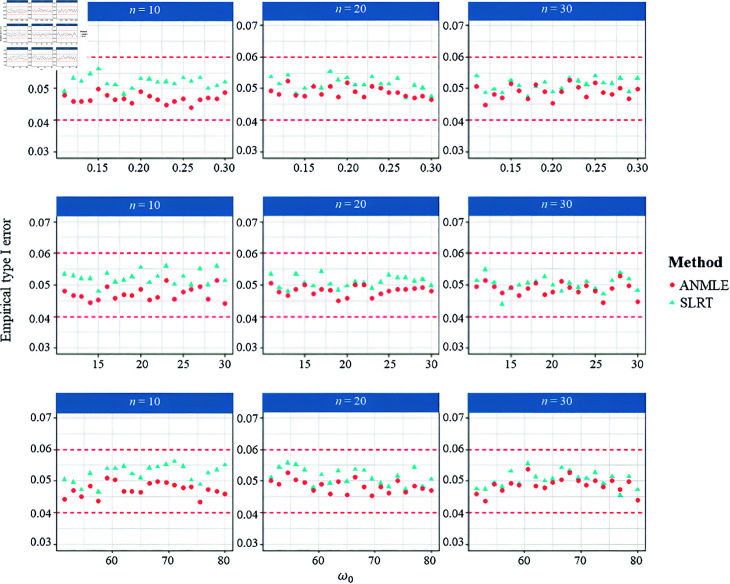
Empirical type I error rates when β=1 and λ=1.5. The figure shows the ability of SLRT and ANMLE methods to control the type I error rate under hypothesis $H_{0}:\omega =\omega_{0}$ versus $H_{1}:\omega \neq \omega_{0}$ for varying values of $\omega_{0}$ and sample sizes n∈{10,20,30}.

**Fig 8:**
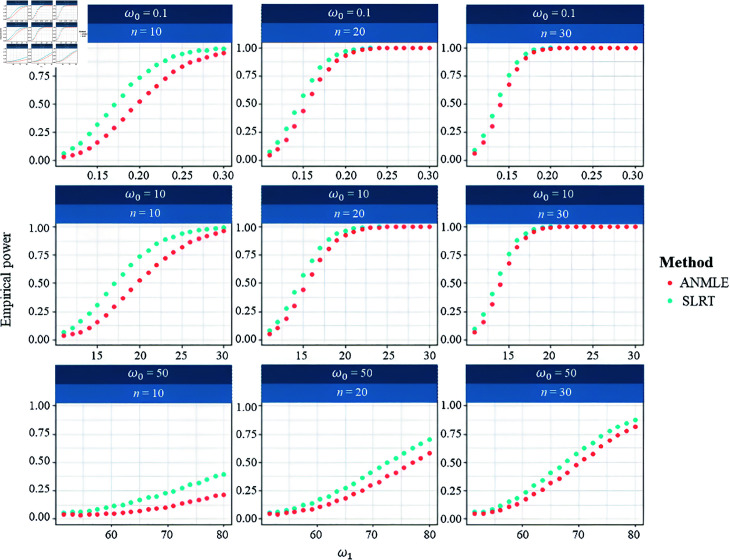
Empirical powers when β=1 and λ=1.5. The figure shows the empirical powers of SLRT and ANMLE methods under hypothesis $H_{0}:\omega =\omega_{0}$ versus $H_{1}:\omega =\omega_{1}$ for varying values of $\omega_{0}$ and $\omega_{1}$, and sample size n∈{10,20,30}.

In addition, we investigated the performance of the proposed method (SLRT) in scenarios involving large values of ω to evaluate whether it remains effective under such conditions. We also performed a sensitivity analysis (see Table 9) to assess the robustness of the proposed method. Regarding Fig 7, the empirical Type I error analysis across varying true null values ($\omega_{0}=0.1,10,50$) and sample sizes (n=10,20,30) reveals that both the SLRT and ANMLE methods maintain proper control of the nominal significance level of 0.05. For the power analysis, the SLRT consistently demonstrates superior power to the ANMLE across all configurations, as shown in Fig 8. This advantage is especially noticeable in small-sample settings and when the deviation between $\omega_{1}$ and $\omega_{0}$ is moderate. As the sample size increases, both methods approach full power under strong alternatives. However, SLRT maintains higher sensitivity throughout various scenarios. These results underscore the robustness of SLRT in detecting deviations from the null hypothesis, particularly when sample sizes are limited or effect sizes are subtle.

**Table 9:** Sensitivity analysis of power for SLRT and ANMLE under varying conditions.

Factor varied	Power trend of ANMLE	Power trend of SLRT	Comparison
Increasing *n*	Substantial increase	Rapid increase	SLRT achieves high power faster
Increasing $\omega_{1}$	Gradual increase; slower	Steeper increase; reaches	SLRT more sensitive to small
	when $\omega_{0}$ is large	power ≈ 1 quickly	changes in $\omega_{1}$
Small *n*	Power low unless $\omega_{1}$ is large	Higher power at small $\omega_{1}$	SLRT outperforms ANMLE
Moderate/	Power converges to 1	Faster convergence to	Performance gap narrows as *n*
Large *n*		power = 1	increases

^*^Sensitivity analysis of power under $H_{0}:\omega =\omega_{0}$ versus $H_{1}:\omega =\omega_{1}$.

Notice that we also examined cases with large β and λ. The results remained consistent, showing that the SLRT still outperforms the ANMLE. As such, these results are omitted for brevity.

## PIW4LIFETIME web application development

In this study, we have developed the PIW4LIFETIME web application using the *Shiny* package in *R* [47], which can be accessible at https://jularatchumnaul.shinyapps.io/PIW4LIFETIME/. PIW4LIFETIME provides a novel platform for data analysis using the Poisson Inverse Weibull (PIW) distribution, a specialized statistical model. The PIW distribution is particularly advantageous for lifetime data analysis, as it offers greater flexibility in modeling a wide range of failure behaviors that are not effectively captured by more commonly used distributions, such as the Weibull or exponential distributions. PIW4LIFETIME assists users in determining whether their data follows the PIW distribution or fits with other lifetime distributions. Moreover, it helps users find the maximum likelihood estimates of the parameters of the PIW distribution and perform a two-sided and one-sided test for the scale parameter of the PIW distribution using the SLRT.

Unlike the extensive support for many lifetime distributions in programming environments like *R*, currently, there is no dedicated package or tool for analyzing the PIW distribution; this lack of resources has led to challenges for researchers and practitioners who want to use the PIW distribution for reliability analysis, survival studies, and risk assessment. Therefore, PIW4LIFETIME was developed to fill this gap, and its key features are shown below.

### Architecture

The PIW4LIFETIME web application was developed using the *R* Shiny framework, enabling interactive web-based data analysis. The backend is built on *R* scripts that handle data input, parameter estimation via maximum likelihood, and hypothesis testing using the SLRT. The front end provides a user-friendly interface for uploading data, selecting test options, and visualizing results.

### Validation procedures

We validated the application by comparing its outputs with results from manually coded R scripts used in our simulation studies. The consistency of parameter estimates, test statistics, and *p*-values across multiple datasets confirms the correctness of the implementation. Additionally, we conducted internal testing using synthetic datasets with known parameters to ensure the accuracy and stability of the SLRT computations.

### Interface usability

The interface is designed to be intuitive for both statisticians and applied researchers. It includes:

- **Data upload:** Users can easily upload their datasets in .csv format for analysis.
- **Data visualization:** PIW4LIFETIME allows users to view various graphs of the PIW distribution, including the probability density function, the cumulative distribution function, the survival function, and the hazard function.
- **Maximum likelihood estimation (MLE):** PIW4LIFETIME estimates the parameters of the PIW distribution (scale, shape, and location) using the MLE method. These estimates are visually highlighted for quick reference.
- **Goodness-of-fit tests and models comparison:** PIW4LIFETIME includes the Anderson-Darling (AD) and Cramér-von Mises (CVM) tests to assess how well the PIW distribution fits the given data. Results are presented with test statistics, *p*-values, and interpretations. Moreover, it also compares the PIW distribution with other common distributions (gamma, Weibull, log-normal, and exponential) using statistical metrics such as AD and CVM statistics, Akaike Information Criterion (AIC), and Bayesian Information Criterion (BIC) to identify the best-fitting model for the given data.
- **Hypothesis testing:** PIW4LIFETIME provides hypothesis testing for the scale parameter (ω) of the PIW distribution using the SLRT. It outputs the test statistic, *p*-value, and a clear conclusion based on the selected significance level.

The users’ manual of the PIW4LIFETIME web application is provided in S2 Appendix.

## Numerical illustrations via PIW4LIFETIME

In this section, we present the analysis using the PIW distribution based on the bladder cancer remission times dataset. This dataset is published in the book *Statistical Methods for Survival Data Analysis* by Elisa T. Lee and John Wenyu Wang [48]. Although the dataset is not available online, it can be accessed through the book, which offers detailed information and context about the data. Researchers interested in this dataset can refer to the book for comprehensive access and understanding.

The remission times (in months) for a random sample of 128 patients with bladder cancer are presented below:

0.08, 2.09, 3.48, 4.87, 6.94, 8.66, 13.11, 23.63, 0.20, 2.23, 3.52, 4.98, 6.97, 9.02, 13.29, 0.40, 2.26, 3.57, 5.06, 7.09, 9.22, 13.80, 25.74, 0.50, 2.46, 3.64, 5.09, 7.26, 9.47, 14.24, 25.82, 0.51, 2.54, 3.70, 5.17, 7.28, 9.74, 14.76, 26.31, 0.81, 2.62, 3.82, 5.32, 7.32, 10.06, 14.77, 32.15, 2.64, 3.88, 5.32, 7.39, 10.34, 14.83, 34.26, 0.90, 2.69, 4.18, 5.34, 7.59, 10.66, 15.96, 36.66, 1.05, 2.69, 4.23, 5.41, 7.62, 10.75, 16.62, 43.01, 1.19, 2.75, 4.26, 5.41, 7.63, 17.12, 46.12, 1.26, 2.83, 4.33, 5.49, 7.66, 11.25, 17.14, 79.05, 1.35, 2.87, 5.62, 7.87, 11.64, 17.36, 1.40, 3.02, 4.34, 5.71, 7.93, 11.79, 18.10, 1.46, 4.40, 5.85, 8.26, 11.98, 19.13, 1.70, 3.25, 4.50, 6.25, 8.37, 12.02, 2.02, 3.31, 4.51, 6.54, 8.53, 12.03, 20.28, 2.02, 3.36, 6.76, 12.07, 21.73, 2.07, 3.36, 6.93, 8.65, 12.63, 22.69 (see S1 File for the data table in .csv format).

To determine whether the remission times follow the PIW distribution, we utilized the proposed web application to perform goodness-of-fit tests. The results show that the dataset adheres the PIW distribution, with Anderson-Darling and Cramér-von Mises test statistics of 1.921 (*p*-value = 0.696) and 0.212 (*p*-value = 0.955), respectively. Furthermore, our analysis found that the fitted PIW cumulative distribution function closely aligns with the empirical CDF, suggesting that the PIW model reasonably fits this dataset.

Using the PIW4LIFETIME web application, we also obtained the parameter estimates for ω, β, and λ through the maximum likelihood method. The resulting estimates are $\hat{\omega }=9.401$, $\hat{\beta }=0.157$, and $\hat{\lambda }=790.555$.

In the context of bladder cancer remission times, which follow the PIW distribution, we tested the hypothesis $H_{0}:\omega =9$ against the alternative hypothesis $H_{1}:\omega \neq 9$ using two methods: the signed log-likelihood ratio test (SLRT) and the asymptotic normality of the maximum likelihood estimators (ANMLE). The SLRT resulted in a test statistic of 0.160 and a *p*-value of 0.837, while the ANMLE produced a test statistic of 1.182 with a *p*-value of 0.237, as summarized in Table 10. Since both *p*-values exceed the 0.05 significance level, we found insufficient evidence to conclude that ω significantly differs from 9. Consequently, both methods suggest that the observed remission times do not provide strong evidence against the null hypothesis. Although both methods led to the same conclusion in this case, our simulation study shows that SLRT generally achieves higher power. Therefore, in scenarios where the two methods yield different conclusions, we recommend prioritizing the SLRT result due to its superior sensitivity and robustness demonstrated in the simulation-based sensitivity analysis.

**Table 10:** Test statistics, *p*-value, and conclusion for testing $H_{0}:\omega =9$ versus $H_{1}:\omega \neq 9$ at the significance level of 0.05.

Method	Statistics	p -value	Conclusion
SLRT	0.160	0.873	Cannot reject *H*_0_
ANMLE	1.182	0.237	Cannot reject *H*_0_

In the PIW distribution, the scale parameter ω governs the dispersion and location of the distribution of the remission time variable *x*. Although ω does not directly represent time, it plays a crucial role in shaping the distribution of remission durations. Testing hypotheses about ω, such as $H_{0}:\omega =9$, allows researchers to evaluate whether the observed data are consistent with a specific assumed scale of the distribution. In the real-world application involving remission times for bladder cancer patients, both the SLRT and ANMLE methods were used to test whether the scale parameter is equal to 9. Since both tests failed to reject the null hypothesis, the results suggest that the remission time data are statistically consistent with a PIW distribution characterized by ω=9. This finding supports the use of this parameter value in modeling and interpreting remission behavior. In practice, such validation can inform clinical expectations, guide follow-up scheduling, and support the development of predictive models for patient outcomes. By confirming that the assumed scale parameter aligns with empirical data, healthcare professionals and researchers can make more informed decisions based on statistically sound models.

The *R* code for the SLRT, which tests the scale parameter of the PIW distribution using the remission times dataset, is provided in S2 File.

## Discussions and conclusions

In this study, we introduce the signed log-likelihood ratio test (SLRT) to perform a hypothesis test for the scale parameter of the Poisson Inverse Weibull (PIW) distribution. Additionally, we compare the performance of the proposed SLRT with the test that relies on the asymptotic normality of the maximum likelihood estimators (ANMLE), focusing on the empirical type I error rate and power.

Our findings illustrated that the SLRT effectively maintained type I error probabilities within acceptable limits across all scenarios at the significance level of 0.05, meeting Cochran’s criterion with error rates between 0.04 and 0.06. Furthermore, our findings showed that the empirical type I error rates of the SLRT remain consistent across various sample sizes and parameter configurations. These results align with the findings of Waguespack *et al*. (2020), demonstrating that type I error rates remain relatively stable across different parameter settings as sample sizes vary [49]. In contrast, the ANMLE method exhibited conservative behavior, with its empirical type I error rates frequently falling below the specified significance level. This finding aligns with the research conducted by Chumnaul and Sepehrifar (2022) [50], which demonstrated that empirical type I error rates for the ANMLE were notably lower than the nominal level, highlighting its conservative nature. However, they noted that as the sample sizes (*n*) increases, the conservatism of the test based on the Fisher information matrix decreases; this result contradicts our findings, where the simulations revealed that increasing the sample size does not reduce the conservative nature of the ANMLE method, indicating that larger sample sizes do not increase its empirical type I error rates.

Regarding the SLRT, our proposed method exhibited high empirical power even with small sample sizes (n=10,15). This finding is consistent with the research by Krishnamoorthy and León-Novelo (2014), who found that the SLRT retained its statistical accuracy and efficiency in small-sample scenarios. This result suggests that the SLRT is robust and reliable for statistical analysis when data are limited. Therefore, it is beneficial for early-stage research or preliminary investigations where sample sizes are often small. On the other hand, the ANMLE exhibited low power in most cases, indicating that it is less sensitive to detecting true effects in smaller samples compared to the SLRT. This lower power may limit its utility when detecting subtle differences or changes, which is crucial, potentially resulting in a higher likelihood of type II errors (failing to reject a false null hypothesis).

Interestingly, when testing the hypothesis $H_{0}:\omega =0.25$ versus $H_{1}:\omega =\omega_{1}$ with medium (n=30,50) and large (n=80,100) sample sizes, the power of the ANMLE begins to approach that of the SLRT. This result is consistent with findings by Chumnaul and Sepehrifar (2022), who reported that the ANMLE exhibits varying power under different scenarios, which is influenced by the scale parameter values being tested. Additionally, we observed that as the scale parameter ω increases, the empirical power of both the SLRT and ANMLE decreases. In contrast, as parameter λ increases, the empirical power of both methods also increases. However, it is important to note that despite improvements with larger sample sizes, the ANMLE never outperforms the SLRT. This consistent underperformance highlights a fundamental limitation of the ANMLE, which may be due to its underlying statistical assumptions or methodological constraints. Thus, while the ANMLE becomes more competitive with larger sample sizes, researchers looking for the most powerful statistical methods may still prefer the SLRT, particularly when detecting the smallest effect.

Notice that, in our simulations, we also performed the likelihood ratio test (LRT) and the score test. However, we observed that the LRT and SLRT methods exhibited identical empirical type I error rates and powers across all scenarios considered. This finding suggests that the additional directional information in SLRT did not affect the test outcome. As a result, we only report the results of the SLRT. We also decided to exclude the score test from our evaluation due to its poor performance; despite its high power, it resulted in unacceptably high type I error rates, making it unsuitable for reliable inference.

In summary, this study introduces the signed log-likelihood ratio test (SLRT) for testing the scale parameter of the Poisson Inverse Weibull (PIW) distribution, comparing its performance with the asymptotic normality of the MLEs (ANMLE) method. Key findings reveal that the SLRT maintains type I error rates within acceptable limits across various scenarios and sample sizes, demonstrating robustness and reliability even with small samples. Moreover, the SLRT consistently exhibits higher empirical power, making it more effective in detecting true effects. In contrast, the ANMLE method shows conservative behavior, with lower empirical type I error rates and reduced power, particularly in smaller samples. Therefore, practical implications highlight the SLRT’s utility in early-stage research and preliminary investigations, where sample sizes are limited, ensuring accurate and efficient statistical analysis.

## Limitation and future work

A limitation of this study is the exclusive focus on the SLRT and ANMLE methods for hypothesis testing without considering the modified signed log-likelihood ratio test (MSLRT). The MSLRT is recognized as a more powerful and robust alternative to the standard SLRT, offering improved accuracy in detecting parameter differences. However, the MSLRT was not included in this analysis due to its significantly higher computational demands and longer processing time, making it impractical within the scope of this study. Specifically, the MSLRT requires extensive processing time, often many hours, to yield results, which is not feasible for the PIW4LIFETIME web application we developed. This web application is designed to provide timely and efficient statistical analysis for lifetime data under the PIW distribution. Therefore, integrating the MSLRT would compromise its operational efficiency.

For future research, several potential extensions of this work could lead to further advancements in statistical analysis. For example, future research could integrate the MSLRT to fully explore its benefits because it might offer insights and more reliable conclusions. Also, optimizing the computational efficiency of the MSLRT could be a promising direction for future work to overcome its current limitations, such as MSLRT algorithmic improvements. Additionally, bootstrap-based SLRT approximations present a compelling alternative for enhancing inference accuracy while managing computational demands. These resampling-based methods can improve small-sample performance without the full complexity of the MSLRT, making them suitable for practical applications where computational resources are limited. Moreover, our research contributes to reliable and efficient statistical analysis by ensuring that the SLRT maintains type I error rates within acceptable limits and exhibits higher empirical power. These improvements in hypothesis testing and parameter estimation can significantly enhance the quality of predictive models built on the PIW distribution. Therefore, future research could build on our findings to develop robust predictive models, leveraging the improved inference methods we have introduced. In addition, extending the PIW distribution to multivariate contexts could also offer deeper insights into lifetime data analysis.

## Supporting information

S1 Appendix Regularity conditions. (PDF)

S2 Appendix PIW4LIFETIME user manual. (PDF)

S1 File Data table of remission times. (CSV)

S2 File R code for the SLRT. (PDF)
