# Supplementary material for: Signed log-likelihood ratio test for the scale parameter of Poisson Inverse Weibull distribution with the development of PIW4LIFETIME web application
Source: PLoS One. 2025 Aug 1;20(8):e0329293. doi: 10.1371/journal.pone.0329293 (PMC12316401; doi:10.1371/journal.pone.0329293)
Supplement: S1 Appendix — (PDF) [file pone.0329293.s001.pdf]

Regularity Conditions

Condition 1: The probability density functions are distinct, that is, for

$$\theta \neq \theta' \Rightarrow f(x;\theta) \neq f(x;\theta').$$

Proof: In the case of the Poisson Inverse Weibull (PIW) distribution, let $X \sim PIW(\varpi, \beta, \lambda)$ with the probability density function (pdf):

$$f(x;\varpi,\beta,\lambda) = \frac{\varpi\beta\lambda}{1-\exp(-\lambda)} x^{-(1+\beta)} \exp[-\lambda \exp(-\varpi x^{-\beta}) - \varpi x^{-\beta}]; \quad x > 0, \ \ \varpi, \beta, \lambda > 0 .$$

Here, we assume

$$f(x;\varpi_1, \beta_1, \lambda_1) = f(x;\varpi_2, \beta_2, \lambda_2)$$

for all *x*. Compare the functional forms:

$$\frac{\varpi_1\beta_1\lambda_1}{1-\exp(-\lambda_1)} x^{-(1+\beta_1)} \exp[-\lambda_1 \exp(-\varpi_1 x^{-\beta_1}) - \varpi_1 x^{-\beta_1}] = \frac{\varpi_2\beta_2\lambda_2}{1-\exp(-\lambda_2)} x^{-(1+\beta_2)} \exp[-\lambda_2 \exp(-\varpi_2 x^{-\beta_2}) - \varpi_2 x^{-\beta_2}]$$

Equate the coefficients and exponents:

i) The terms involving *x* must match, leading to $\beta_1 = \beta_2$.

ii) The coefficients must match, leading to $\varpi_1 = \varpi_1$ and $\lambda_1 = \lambda_1$.

Thus, the parameters ϖ, β, and λ uniquely identify the pdf.

Condition 2: The probability density functions have common support for all θ.

Proof: In the case of the PIW distribution, the support of the pdf is the set of x values for which $f(x;\varpi,\beta,\lambda) > 0$. For the given pdf, the support is $x > 0$, which does not depend on the parameters $\varpi, \beta,$ or λ.

Condition 3: The point θ, the real parameter that is, is an interior point in some set Ω.

Proof: The parameter $\theta = (\varpi, \beta, \lambda)$ must be an interior point in some set Ω. Since $\varpi > 0$, $\beta > 0$, and $\lambda > 0$, θ lies in the positive orthant of $\mathbb{R}^3$, which is an open set. Therefore, θ is an interior point.

Condition 1-3 guarantee that the likelihood is maximized at the true parameter θ_0 and then that the maximum likelihood estimator $\hat{\theta}$ that solves the equation $\frac{\partial l(\theta)}{\partial \theta} = 0$ is consistent.

Condition 4: The probability density function $f(x;\theta)$ is twice differentiable as a function of θ.

Proof: In the case of the PIW distribution, we can obtain the partial derivatives of $f(x;\varpi,\beta,\lambda)$ with respect to ϖ, β, λ, treating *x* as a constant, as follows:

$$\frac{\partial}{\partial \varpi} f(x;\varpi,\beta,\lambda) = \frac{\beta\lambda}{1-\exp(-\lambda)} x^{-(1+\beta)} \exp[-\lambda\exp(-\varpi x^{-\beta}) - \varpi x^{-\beta}] +$$
$$\frac{\varpi\beta\lambda}{1-\exp(-\lambda)} x^{-(1+\beta)} \exp[-\lambda\exp(-\varpi x^{-\beta}) - \varpi x^{-\beta}] x^{-\beta}(\lambda\exp(-\varpi x^{-\beta}) - 1)$$
$$\frac{\partial}{\partial \beta} f(x;\varpi,\beta,\lambda) = \frac{\varpi\lambda}{1-\exp(-\lambda)} x^{-(1+\beta)} \exp[-\lambda\exp(-\varpi x^{-\beta}) - \varpi x^{-\beta}] -$$
$$\frac{\varpi\beta\lambda}{1-\exp(-\lambda)} x^{-(1+\beta)} \exp[-\lambda\exp(-\varpi x^{-\beta}) - \varpi x^{-\beta}] \ln x +$$
$$\frac{\varpi\beta\lambda}{1-\exp(-\lambda)} x^{-(1+\beta)} \exp[-\lambda\exp(-\varpi x^{-\beta}) - \varpi x^{-\beta}] \varpi x^{-\beta} \ln x(\lambda\exp(-\varpi x^{-\beta}) + 1)$$
$$\frac{\partial}{\partial \lambda} f(x;\varpi,\beta,\lambda) = \varpi\beta \frac{(1-\exp(-\lambda)) - \lambda\exp(-\lambda)}{(1-\exp(-\lambda))^2} x^{-(1+\beta)} \exp[-\lambda\exp(-\varpi x^{-\beta}) - \varpi x^{-\beta}] -$$
$$\frac{\varpi\beta\lambda}{1-\exp(-\lambda)} x^{-(1+\beta)} \exp[-\lambda\exp(-\varpi x^{-\beta}) - \varpi x^{-\beta}] \exp(-\varpi x^{-\beta})$$

The second partial derivatives $\frac{\partial^2 f}{\partial \varpi^2}, \frac{\partial^2 f}{\partial \beta^2}, \frac{\partial^2 f}{\partial \lambda^2}, \frac{\partial^2 f}{\partial \varpi \partial \beta}, \frac{\partial^2 f}{\partial \varpi \partial \lambda}, \frac{\partial^2 f}{\partial \beta \partial \lambda}$ can also be obtained based on the first derivatives, but we do not show the derivations here. The expressions of second partial derivatives are algebraically heavy but can be simplified numerically. Here, we can see that the first and second derivatives exist and are continuous, ensuring that $f(\varpi,\beta,\lambda)$ is twice differentiable.

Condition 5: The integral $\int_{-\infty}^{\infty} f(x;\theta)dx$ can be differentiated twice under the integral sign as a function of θ.

Proof: In the case of the PIW distribution, we have to show that

$$\frac{\partial^2}{\partial\theta_i \partial\theta_j} \int_0^{\infty} f(x;\theta)dx = \int_0^{\infty} \frac{\partial^2 f(x;\theta)}{\partial\theta_i \partial\theta_j} dx$$

for all $\theta \in \{\varpi,\beta,\lambda\}$, and for all i, j, i.e. interchanging differentiation and integration is valid.

First, we can see that the PIW pdf is infinitely differentiable in all parameters ϖ,β,λ, and for $x > 0$, it is smooth due to:

i) $x^{-(1+\beta)}$ and $x^{-\beta}$ are smooth for $x > 0$
ii) Composition of exponentials and products of smooth functions
iii) No singularities for $\varpi,\beta,\lambda > 0$

Thus, differentiability of $f(x;\varpi,\beta,\lambda)$ up to the second order is guaranteed.

Second, the exponential decay in the term $\exp(-\varpi x^{-\beta})$ ensures that the tails of the function decay rapidly as $x \to 0^+$ and $x \to \infty$, which helps in bounding the derivatives. We now verify the existence of a dominating function *g(x)* such that:

$$\left| \frac{\partial^2 f(x;\theta)}{\partial\theta_i \partial\theta_j} \right| \le g(x), \quad \text{for all } \theta \in \Theta.$$

This is typically achieved by bounding the derivatives with inequalities and demonstrating that the resulting function is integrable over the interval (0, ∞). Given the presence of exponential decay and polynomial terms, a function *g(x)* can be constructed, although its exact form may be complex.

Since $f(x;\theta)$ is twice differentiable in *θ*, and the second derivatives are continuous and dominated by an integrable function *g*(*x*), Condition 5 is then satisfied.

Condition 4-5 are required to derive the Fisher Information which plays a central role in the theory of convergence of the maximum likelihood estimator.

Condition 6: The pdf $f(x;\theta)$ is 3-time differentiable with respect to *θ*. Further for all *θ* ∈ *Ω*, there exists a constant *c* and a function *M*(*x*) such that

$$\left|\frac{\partial^3 \log f(x;\theta)}{\partial\theta^3}\right| \le M(x),$$

with $E_{\theta_0}[M(x)] < \infty$ for all $|\theta-\theta_0| < c$ and all *x* in the support of *X*.

Proof: The log-pdf of PIW distribution is

$$\log f(x;\varpi,\beta,\lambda) = \log(\varpi) + \log(\beta) + \log(\lambda) - \log(1-e^{-\lambda}) - (\beta+1)\log x - \lambda e^{-\varpi x^{-\beta}} - \varpi x^{-\beta}.$$

All the terms are compositions of smooth functions (log, exponential, polynomials), and so $\log f(x;\varpi,\beta,\lambda) \in C^3$. Therefore, $\log f(x;\varpi,\beta,\lambda)$ is 3-time differentiable with respect to all parameters in the interior of the parameter space.

From the log-pdf, we obtain the third derivative of the log-pdf with respect to ϖ as follows:

$$\frac{\partial^3 \log f(x;\varpi,\beta,\lambda)}{\partial\varpi^3} = \frac{2}{\varpi^3} + \lambda x^{-3\beta} e^{-\varpi x^{-\beta}}.$$

Here, the third derivative $\frac{\partial^3 \log f(x;\varpi,\beta,\lambda)}{\partial\varpi^3}$ is dominated by terms of the form $x^{-3\beta}e^{-\varpi x^{-\beta}}$ because $x^{-\beta} \to 0$ as $x \to \infty$, $x^{-\beta} \to \infty$ as $x \to 0^+$, but $e^{-\varpi x^{-\beta}}$ decays rapidly at both ends. Then for all $\theta \in B(\theta_0, c)$, we can find a function $M(x) = C_1 x^{-k_1} e^{-C_2 x^{-k_2}} + C_3$ for suitable constants $C_i > 0$, such that:

$$\left|\frac{\partial^3 \log f(x;\theta)}{\partial\varpi^3}\right| \le M(x).$$

Similar bounding applies to third derivatives with respect to *β* and *λ* as well, because they also appear inside exponentials and polynomial terms that are dominated for small and large *x*. Therefore, the expectation

$$E_{\theta_0}[M(x)] = \int_0^\infty M(x) f(x;\theta_0) dx$$

is finite.
