# Supplementary material for: Signed log-likelihood ratio test for the scale parameter of Poisson Inverse Weibull distribution with the development of PIW4LIFETIME web application
Source: PLoS One. 2025 Aug 1;20(8):e0329293. doi: 10.1371/journal.pone.0329293 (PMC12316401; doi:10.1371/journal.pone.0329293)
Supplement: S2 Appendix — (PDF) [file pone.0329293.s002.pdf]

# PIW4LIFETIME User Manual

## 1. Introduction

The Poisson Inverse Weibull for Lifetime Data (PIW4LIFETIME) web application was developed using the *Shiny* package in R and is freely accessible at https://jularatchumnaul.shinyapps.io/PIW4LIFETIME/. PIW4LIFETIME offers a unique platform for data analysis utilizing the Poisson Inverse Weibull (PIW) distribution, a specialized statistical model. This distribution is particularly beneficial for analyzing lifetime data, as it provides greater flexibility in modeling a wide range of failure behaviors that may not be effectively captured by more commonly used distributions, such as the Weibull or exponential distributions.

The PIW4LIFETIME application aids users in determining whether their data aligns with the PIW distribution or fits other lifetime distributions. Additionally, it helps users find the maximum likelihood estimates for the parameters of the PIW distribution and perform both two-sided and one-sided tests for the scale parameter using the signed log-likelihood ratio test (SLRT).

## 2. Features of the PIW4LIFETIME web application

The PIW4LIFETIME web application is designed to provide comprehensive tools for analyzing the PIW distribution. It offers a user-friendly interface for uploading data, visualizing distribution functions, estimating parameters, and performing a hypothesis test for the scale parameter. Below are the key features of the application:

**2.1 Data upload:** Users can easily upload their datasets in .csv format for analysis.

**2.2 Data visualization:** PIW4LIFETIME allows users to view various graphs for the PIW distribution, including the probability density function (PDF), the cumulative distribution function (CDF), the survival function, and the hazard function.

**2.3 Maximum likelihood estimation (MLE):** PIW4LIFETIME estimates the parameters of the PIW distribution (scale, shape, and location) using the MLE method. These estimates are visually highlighted for quick reference.

**2.4 Goodness-of-fit tests and models comparison:** PIW4LIFETIME includes the Anderson-Darling (AD) and Cramér-von Mises (CVM) tests to assess how well the PIW distribution fits the given data. Results are presented with test statistics, *p*-values, and interpretations. Moreover, it also compares the goodness-of-fit tests of the PIW distribution with other distributions (gamma, Weibull, log-normal, and exponential) using statistical metrics such as AD and CVM statistics, Akaike Information Criterion (AIC), and Bayesian Information Criterion (BIC) to identify the best-fitting model for the given data.

**2.5 Hypothesis testing:** PIW4LIFETIME provides hypothesis testing for the scale parameter ($\omega$) of the PIW distribution using the SLRT. It outputs the test statistics, *p*-value, and a clear conclusion based on the selected significance level.

## 3. Performance of PIW4LIFETIME through an example

The PIW4LIFETIME web application consists of two main functions. Below is an overview of its functionality and usage.

### 3.1 Visualizations of the PIW distribution functions

This function allows users to explore the characteristics of the Poisson Inverse Weibull (PIW) distribution through the "PIW characteristics" tab. Users can input the parameters of the PIW distribution, which include the scale parameter ($\omega$), shape parameter ($\beta$), and location parameter ($\lambda$).

Upon entering these values, PIW4LIFETIME will instantly generate real-time visualizations of the following distribution functions: the probability density function (PDF), cumulative density function (CDF), survival function, and hazard function. Users can observe how variations in the parameter values affect the shapes of these functions, providing a deeper understanding of the behavior of the PIW distribution.

### 3.2 Inferential statistics

This function allows users to find the maximum likelihood estimates for the parameter of the PIW distribution, perform goodness-of-fit tests, and perform hypothesis testing for the scale parameter of the PIW distribution. For this function, users are able to upload their own data for analysis.

In this section, we present the performance of PIW4LIFETIME in data analysis using a real-world dataset. The dataset contains remission times (in months) for a random sample of 128 bladder cancer patients. The remission times are as follows:

0.08, 2.09, 3.48, 4.87, 6.94, 8.66, 13.11, 23.63, 0.20, 2.23, 3.52, 4.98, 6.97, 9.02, 13.29, 0.40, 2.26, 3.57, 5.06, 7.09, 9.22, 13.80, 25.74, 0.50, 2.46, 3.64, 5.09, 7.26, 9.47, 14.24, 25.82, 0.51, 2.54, 3.70, 5.17, 7.28, 9.74, 14.76, 26.31, 0.81, 2.62, 3.82, 5.32, 7.32, 10.06, 14.77, 32.15, 2.64, 3.88, 5.32, 7.39, 10.34, 14.83, 34.26, 0.90, 2.69, 4.18, 5.34, 7.59, 10.66, 15.96, 36.66, 1.05, 2.69, 4.23, 5.41, 7.62, 10.75, 16.62, 43.01, 1.19, 2.75, 4.26, 5.41, 7.63, 17.12, 46.12, 1.26, 2.83, 4.33, 5.49, 7.66, 11.25, 17.14, 79.05, 1.35, 2.87, 5.62, 7.87, 11.64, 17.36, 1.40, 3.02, 4.34, 5.71, 7.93, 11.79, 18.10, 1.46, 4.40, 5.85, 8.26, 11.98, 19.13, 1.70, 3.25, 4.50, 6.25, 8.37, 12.02, 2.02, 3.31, 4.51, 6.54, 8.53, 12.03, 20.28, 2.02, 3.36, 6.76, 12.07, 21.73, 2.07, 3.36, 6.93, 8.65, 12.63, 22.69.

To begin, users need to prepare a data table in .csv format. Next, they should select a method to analyze the data. The following information outlines how to use PIW4LIFETIME for various statistical inference purposes.

**PIW parameters estimates:**

To obtain the maximum likelihood estimates (MLE) of the parameters for the PIW distribution, users must choose the “PIW parameter estimates” option from the *Inferential Statistics* panel. Then, users must upload their prepared data table to PIW4LIFETIME by clicking the "Browse..." button and specifying the variable for analysis. PIW4LIFETIME will quickly identify the maximum likelihood estimates (MLEs) of the parameters of the PIW distribution and produce a probability density function plot based on these parameter estimates.

According to the results from PIW4LIFETIME, the MLEs for parameters $\omega$, $\beta$, and $\lambda$ are 9.4, 0.157, and 789.993, respectively.

**Goodness-of-fit tests:**

To determine whether this dataset follows the PIW distribution, users must choose the “Goodness-of-fit tests” option from the *Inferential Statistics* panel. Then, users must upload their prepared data table to PIW4LIFETIME by clicking the "Browse..." button, specifying the variable for analysis, and setting the significance level. PIW4LIFETIME will evaluate how well the data fits the PIW distribution using the Anderson-Darling and Cramér-von Mises tests. PIW4LIFETIME also displays the Empirical CDF and Fitted PIW CDF graphs to help users

visualize the distribution of the given data compared to the PIW distribution. Moreover, PIW4LIFETIME also compares the goodness-of-fit tests of the PIW distribution with other distributions (gamma, Weibull, log-normal, and exponential) using statistical metrics such as AD and CVM statistics, Akaike Information Criterion (AIC), and Bayesian Information Criterion (BIC) to identify the best-fitting model for the given data.

Regarding results obtained from PIW4LIFETIME, it can be seen that the given dataset adheres to the PIW distribution, with Anderson-Darling and Cramér-von Mises test statistics of 1.921 ($p$-value = 0.696) and 0.212 ($p$-value = 0.955), respectively. Furthermore, it demonstrates that the fitted PIW cumulative distribution function (CDF) closely aligns with the empirical CDF, suggesting that the PIW model reasonably fits this dataset. Notice that the given dataset also fits well with the exponential distribution, as indicated by the smallest Akaike Information Criterion (AIC).

**Hypothesis test for scale parameter:**

To perform a hypothesis test for the scale parameter of the PIW distribution, users must choose the “Hypothesis test for scale parameter” option from the *Inferential Statistics* panel. Then, users must upload their prepared data table to PIW4LIFETIME by clicking the "Browse..." button, specifying the variable for analysis, the hypothesized value, and setting the significance level. Here, we are interested in testing the hypothesis $H_0$: $\omega = 9$ against the alternative hypothesis $H_1$: $\omega \neq 9$ at the significance level of 0.05. Once all the required details are entered into PIW4LIFETIME, it automatically performs the SLRT and provides the user with the test results.

Regarding results obtained from PIW4LIFETIME, it indicates that the SLRT produced a test statistic of 0.160, with a corresponding $p$-value of 0.837. This outcome suggests that the data does not provide strong evidence against the null hypothesis at the significance level of 0.05.
