# Supplementary material for: Signed log-likelihood ratio test for the scale parameter of Poisson Inverse Weibull distribution with the development of PIW4LIFETIME web application
Source: PLoS One. 2025 Aug 1;20(8):e0329293. doi: 10.1371/journal.pone.0329293 (PMC12316401; doi:10.1371/journal.pone.0329293)
Supplement: S2 File — (PDF) [file pone.0329293.s004.pdf]

R Code for the SLRT

```
datapiw <- S1_File
data <- data.frame(datapiw)

piw_pdf <- function(x, omega, lambda, beta) {
  term1 <- (omega * beta * lambda) / (1 - exp(-lambda))
  term2 <- x^(-(1 + beta))
  term3 <- exp(-lambda * exp(-omega * x^(-beta)))
  term4 <- exp(-omega * x^(-beta))
  return(term1 * term2 * term3 * term4)
}

log_likelihood1 <- function(params, data) {
omega <- params[1]
lambda <- params[2]
beta <- params[3]

log_pdf <- log((omega * beta * lambda) / (1 - exp(-lambda))) +
log(data^(-(1 + beta))) +
-lambda * exp(-omega * data^(-beta)) +
-omega * data^(-beta)

sum_log_pdf <- sum(log_pdf)
return(sum_log_pdf)
}

test <- 9
omega <- test

log_likelihood2 <- function(params, data) {
  lambda <- params[1]
  beta <- params[2]

  log_pdf <- log((omega * beta * lambda) / (1 - exp(-lambda))) +
    log(data^(-(1 + beta))) +
    -lambda * exp(-omega * data^(-beta)) +
    -omega * data^(-beta)

  sum_log_pdf <- sum(log_pdf)
  return(sum_log_pdf)
}
```

```
neg_log_likelihood1 <- function(params, data) {
  -log_likelihood1(params, data)
}

neg_log_likelihood2 <- function(params, data) {
  -log_likelihood2(params, data)
}

initial_values <- c(omega = 0.5, lambda = 0.5, beta = 0.5)
initial_values2 <- c(lambda = 0.5, beta = 0.5)

data <- data$PIW_data

result1 <- optim(initial_values, neg_log_likelihood1, data = data, method = "L-BFGS-B",
lower = c(0.001, mean(data), 0.001), upper = c(Inf, Inf, Inf), control = list(maxit = 10000))
result2 <- optim(initial_values2, neg_log_likelihood2, data = data, method = "L-BFGS-B",
lower = c(mean(data), 0.001), upper = c(Inf, Inf), control = list(maxit = 10000))

estimated_params1 <- result1$par
estimated_params2 <- result2$par
names(estimated_params1) <- c("omega", "lambda","beta")
names(estimated_params2) <- c("lambda","beta")

omega_mle1 <- estimated_params1["omega"]
lambda_mle1 <- estimated_params1["lambda"]
beta_mle1 <- estimated_params1["beta"]
lambda_mle2 <- estimated_params2["lambda"]
beta_mle2 <- estimated_params2["beta"]

n <- nrow(PIW_data.cancer)

lnmle <- n*log(omega_mle1*lambda_mle1*beta_mle1) - n*log(1-exp(-lambda_mle1)) -
(beta_mle1 + 1)*sum(log(data)) - omega_mle1*sum(data^(-beta_mle1)) -
lambda_mle1*sum(exp(-omega_mle1*data^(-beta_mle1)));
lnh0 <- n*log(test*lambda_mle2*beta_mle2) - n*log(1-exp(-lambda_mle2)) - (beta_mle2 +
1)*sum(log(data)) - test*sum(data^(-beta_mle2)) - lambda_mle2*sum(exp(-test*data^(-
beta_mle2)));

lr <- -2*(lnh0-lnmle)
slrt <- sign(omega_mle1 - test)*sqrt(lr)
ptest <- pnorm(abs(slrt), lower.tail=TRUE)
pval <- 2*(1-ptest)

conflevel1 <- 0.05
if (pval > conflevel1){
```

```
  cat(paste0(" --------------------------------------------------------", "\n"),
      paste0( "Hypothesis test for the scale parameter using SLRT method", "\n"),
      paste0("--------------------------------------------------------", "\n"),
      paste0("H0: The scale parameter is equal to ", test, "\n"),
      paste0("H1: The scale parameter is not equal to ", test, "\n", "\n"),
      paste0("Test statistic: ", format(round(slrt, 3), nsmall = 3), "\n"),
      paste0("p-value: ", format(round(pval, 3), nsmall = 3), "\n"),
      paste0("Conclusion: The scale parameter is equal to ", test, "at the", "\n"),
      paste0(conflevel1*100,"% significance level.", "\n"))
}else if (pval <= conflevel1){
  cat(paste0(" --------------------------------------------------------", "\n"),
      paste0( "Hypothesis test for the scale parameter using SLRT method", "\n"),
      paste0("--------------------------------------------------------", "\n"),
      paste0("H0: The scale parameter is equal to ", test, "\n"),
      paste0("H1: The scale parameter is not equal to ", test, "\n", "\n"),
      paste0("Test statistic: ", format(round(slrt, 3), nsmall = 3), "\n"),
      paste0("p-value: ", format(round(pval, 3), nsmall = 3), "\n"),
      paste0("Conclusion: The scale parameter is not equal to ", test, "at the", "\n"),
      paste0(conflevel1*100,"% significance level.", "\n"))
}
```
